# Design, synthesis and photochemical properties of the first examples of iminosugar clusters based on fluorescent cores

**DOI:** 10.3762/bjoc.11.74

**Published:** 2015-05-06

**Authors:** Mathieu L Lepage, Antoine Mirloup, Manon Ripoll, Fabien Stauffert, Anne Bodlenner, Raymond Ziessel, Philippe Compain

**Affiliations:** 1 Laboratoire de Synthèse Organique et Molécules Bioactives (SYBIO), Université de Strasbourg/CNRS (UMR 7509), Ecole Européenne de Chimie, Polymères et Matériaux (ECPM), 25 rue Becquerel, 67087 Strasbourg, France; 2 Institut de Chimie et Procédés pour l’Energie, l’Environnement et la Santé (ICPEES), Laboratoire de Chimie Organique et Spectroscopie Avancées (LCOSA), Université de Strasbourg/CNRS (UMR 7515), Ecole Européenne de Chimie, Polymères et Matériaux (ECPM), 25 rue Becquerel, 67087 Strasbourg, France; 3 Institut Universitaire de France, 103 Bd Saint-Michel, 75005 Paris, France

**Keywords:** BODIPY, fluorescent probes, iminosugars, 4-methylumbelliferone, multivalency, pyrene

## Abstract

The synthesis and photophysical properties of the first examples of iminosugar clusters based on a BODIPY or a pyrene core are reported. The tri- and tetravalent systems designed as molecular probes and synthesized by way of Cu(I)-catalysed azide–alkyne cycloadditions are fluorescent analogues of potent pharmacological chaperones/correctors recently reported in the field of Gaucher disease and cystic fibrosis, two rare genetic diseases caused by protein misfolding.

## Introduction

**Figure 1:**
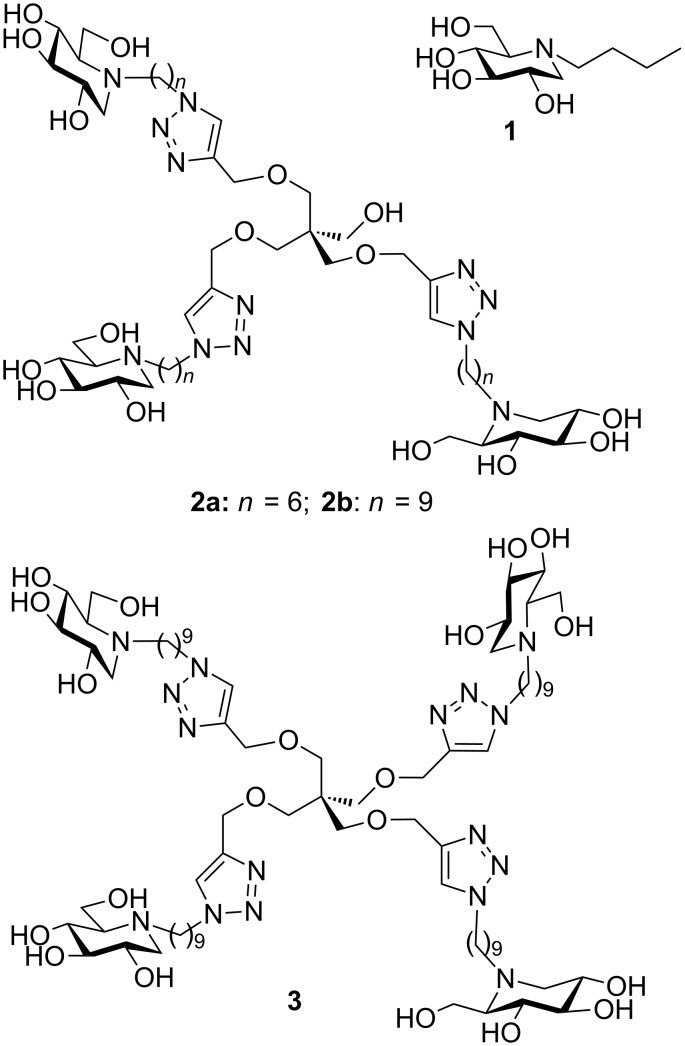
*N*-Bu DNJ (**1**) and examples of potent multivalent pharmacological chaperones and CFTR correctors (**2** and **3**).

Since the isolation in the 1970’s of 1-deoxynojirimycin (DNJ) from natural sources and the finding of its biological activity as an α-glucosidase inhibitor, thousands of sugar mimetics with a nitrogen atom replacing the endocyclic oxygen have been reported in the literature [1–2]. Iminosugars are mainly known to be inhibitors of a number of carbohydrate-processing enzymes with an emphasis on glycosidases [1–2]. In the early 2000’s, iminosugars were, remarkably, found to inhibit metalloproteinases [3], protein kinases [4] and cholinesterases [5], which are enzymes that act on non-sugar substrates. The versatility of iminosugars as inhibitors of enzymes of therapeutic interest has been harnessed to cure a diversity of diseases including diabetes, viral infection, lysosomal storage disorders, tumour metastasis and cystic fibrosis [1]. First therapeutic successes have been obtained as demonstrated by the number of structures involved in clinical trials and two medicines on the market: Glyset (*N*-hydroxyethyl DNJ) for the treatment of complications associated with type II diabetes and Zavesca (*N*-Bu DNJ, **1**), the first oral treatment for Gaucher and Niemann–Pick diseases (Figure 1) [1,6–8]. Despite their high therapeutic potential, the extensive studies in the field and the myriad of compounds synthesized, very few examples of multivalent iminosugars were reported in the literature until recently [9–10]. From 2010, the field has however experienced a major take-off with the discovery of the first strong multivalent effects in glycosidase inhibition observed with DNJ clusters based on β-cyclodextrin or C_60_ cores showing strong affinity enhancements over the corresponding monomers (up to 610-fold per DNJ unit) [11–12]. In the following years, an impressive ever-growing number of multivalent iminosugars based on various scaffolds, ligands and linkers have been synthesized to further investigate the impact of multivalency on glycosidase inhibition [9–26]. The interest of the inhibitory multivalent effect for drug discovery was demonstrated by targeting glycosidases involved in rare genetic diseases linked to misfolded proteins [24–26]. The first examples of multivalent iminosugars such as **2** and **3** acting as pharmacological chaperones were thus disclosed in the field of Gaucher disease, the most common lysosomal storage disorder (Figure 1) [24–25]. DNJ clusters **2** and **3** are indeed able to increase mutant β-glucocerebrosidase (GCase) residual activity levels as much as 3.3-fold in cells of Gaucher patients at micromolar concentrations. In another rare genetic disease, the rescue by multimeric correctors of the mutant CFTR protein implied in cystic fibrosis led to the first report of a multivalent effect for amending protein folding defects in cells [26]. As judged by EC_50_ (half-maximal effective concentration) values, trivalent DNJ clusters **2** were indeed up to 225-fold more efficient as CFTR correctors than the clinical candidate *N*-Bu DNJ (**1**), a potent inhibitor of trimming ER glucosidases [26]. Taken together, these recent studies provide new therapeutic answers for a number of protein folding disorders [27–28] but also raise many fundamental questions concerning the mechanisms at play. In the present paper, we report the first examples of fluorescently-labeled multivalent iminosugars designed as molecular tools to investigate the mode of action of pharmacological chaperones/correctors in cells and in vivo, and get insights into the multivalent effect observed in CFTR correcting activity. The originality of our approach relies on the fact that, in the structures designed, this is the scaffold itself [29–30], based on a pyrene or a boron-dipyrromethene (F-BODIPY) dye, which has fluorescence activity.

## Results and Discussion

### Synthetic design

**Figure 2:**
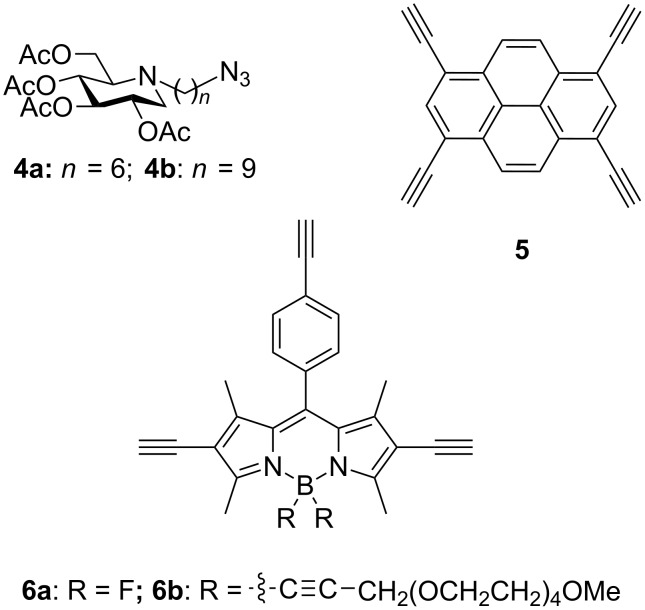
Azide-armed DNJ derivatives **4** and polyalkyne “clickable” scaffolds **5** and **6**.

The fluorescent probes were designed as analogues of the best multivalent pharmacological chaperones/correctors reported so far that typically display three to four copies of a DNJ ligand linked to a central core via an alkyl chain spacer (Figure 1) [24–26]. The choice of the fluorophore core is naturally primordial for the design of photostable, water-soluble and biocompatible probes with the required photophysical properties. An additional challenge is that, as the central core of a multivalent system, the fluorophore structure defines also its valency, size and shape. Difluoroboradiaza-*s*-indacenes, commonly named boron-dipyrromethene dyes (F-BODIPY), were logically selected for the construction of the probes. These compounds indeed combine high fluorescence quantum yields and high molar extinction coefficients, strong chemical and photochemical stability in solution and in solid state. In addition, they can be easily derivatized [31–37]. If the optical properties of BODIPY are very sensitive to modification of the pyrrole core [38–39], they are less sensitive to the substitution of the central pseudo *meso* position [40–41]. Additionally, the fluorine substitution at the boron has less influence on the spectroscopic properties of the dyes [42]. So far, major endeavors have been dedicated to the preparation of classical F-BODIPY structures and less common E-BODIPY (E for ethynyl) and the examination of their spectroscopic and salient physical properties [43–47]. We have recently argued the case that the fluoro-substitution of boraindacene was a mean to considerably increase the solubility, the stability and the steric hindrance avoiding the formation of aggregates [48]. In some cases, special sensing properties [49] may be induced by adequate tailoring, including fluorescence amplification [50], and ratiometric pH reporter for imaging protein–dye conjugates in living cells [51], or display physiological binding of D-glucose [52]. The pyrene nucleus was also selected as an alternative fluorophore since it may be easily tetrafunctionalized at the 1, 3, 6 and 8 positions to give a suitable core for the synthesis of tetravalent clusters [53]. In addition, this fluorophore was chosen for its biological/chemical stability and its photophysical properties including high extinction coefficient with reliable fluorescence [54–55]. Another interest of the pyrene scaffold lies in its rigidity, a property that may favourably impact inhibitory multivalent effects [9,11,16,19]. A convergent approach comprising the attachment of azide-armed iminosugars **4** [11–12] on polyalkyne “clickable” scaffolds **5** and **6** via Cu(I)-catalyzed azide–alkyne cycloaddition (CuAAC) was performed for achieving our synthetic goals (Figure 2) [56–57]. With the objective of increasing water solubility and chemical stability in biological medium, triyne **6b**, an analogue of F-BODIPY-based scaffold **6a** was prepared by replacing the fluoro groups on the boron center with ethynyl tetra(ethylene glycol)methyl groups [58–59].

### Synthesis of the BODIPY precursors

**Scheme 1:**
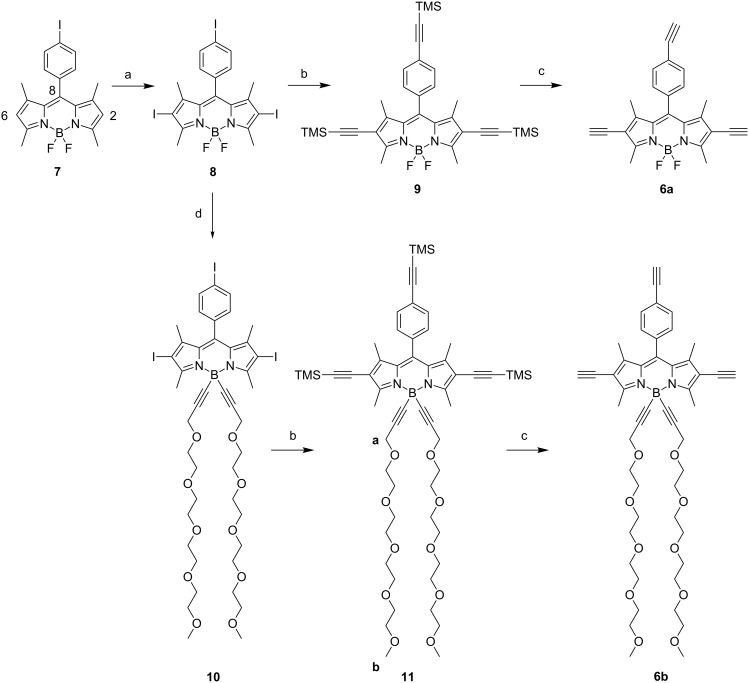
Synthesis of trisubstituted BODIPY derivatives. (a) ICl, CHCl_3_/MeOH, rt, 15 min, quantitative; (b) 3-ethynyltrimethylsilane, [Pd(PPh_3_)_2_Cl_2_], CuI, triethylamine, THF, 16 h, 60 °C, 88% (**9**), 95% (**11**); (c) K_2_CO_3_, DCM/MeOH/water, 50 °C, 16 h, 72% (**6a**), 88% (**6b**); (d) 1-[2”’-(2”-{2’-(2-methoxyethoxy)ethoxy}ethoxy)ethoxy]prop-2-yne, EtMgBr, THF, 60 °C, 16 h, 61%.

The synthesis of the tris-iodo functionalized BODIPY dyes and their acetylenic derivatives is sketched in Scheme 1. The synthesis of derivatives **7** and **8** have previously been reported using a regioselective iodination reaction positions 2 and 6 of the BODIPY [60]. Substitution of both fluoro groups on the boron was realized using the Grignard reagent of 1-[2”’-(2”-{2’-(2-methoxyethoxy)ethoxy}ethoxy)ethoxy]prop-2-yne [61] and the BODIPY derivative **8**. With these precursors in hands it was easy to transform the iodo function to yield the trimethylsilylacetylene derivatives **9** and **11** using standard Sonogashira–Hagihira cross-coupling reactions promoted by low valent palladium precursors [62]. Excellent yields were obtained for the trisubstituted derivatives (88 to 95%). Two diagnostic NMR signals of the poly(ethylene glycol) chains at 4.16 ppm (protons a, integration 4H) and at 3.65 ppm for the methoxy groups (protons b, integration 6H) in addition to the presence of two TMS singlets at 0.20 and 0.28 ppm (respective integration 18 and 9H) confirmed the substitution. Finally, deprotection of the trimethylsilyl group using mild basic conditions provided the target compounds **6a** and **6b** in good yields. Terminal alkynes located in the 2,6 positions were found to resonate at 3.32 ppm and the one in the pseudo meso position 8 resonates at 3.20 ppm.

### Fluorescent DNJ cluster synthesis

**Scheme 2:**
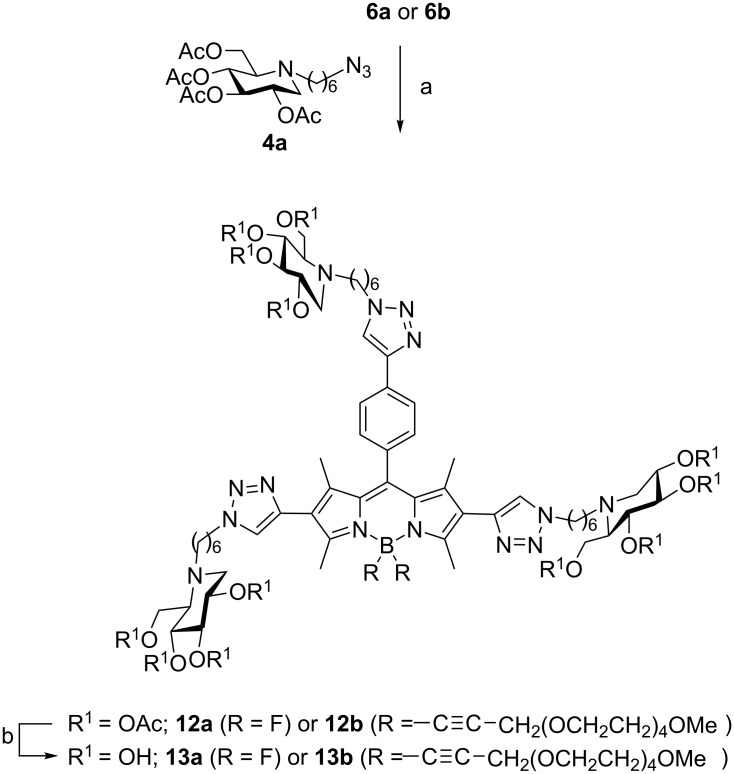
Synthesis of DNJ clusters **13**: (a) CuSO_4_·5H_2_O cat., sodium ascorbate, THF/H_2_O (1:1), 83% (**12a**), 56% (**12b**); (b) Amberlite IRA 400 (OH^−^), MeOH/H_2_O (1:1), rt, quant. (**13a**), quant. (**13b**).

Following a robust strategy developed in our group [11–12], the last stages of the multivalent probe synthesis involved the attachment of peracetylated azido iminosugars **4** on the scaffolds via CuAAC reaction and afterwards *O*-deacetylation using an anion exchange resin. First attempts to perform CuAAC reactions with triyne substrate **6b** bearing a tetraethylene glycol chain tethered to the boron center via an ethynyl bond proved difficult. The use of copper(I) bromide dimethyl sulfide complex [63] at room temperature led to a complex mixture of products. Better results were obtained with copper(II) sulfate and sodium ascorbate under carefully degassed conditions and the desired protected cluster **12b** could be obtained in 56% yield after purification on silica gel (Scheme 2). The major side-product observed which could not be isolated in pure form may correspond to CuAAC reaction of the azido iminosugar **4a** with the terminal alkyne resulting from the cleavage of the carbon–boron bond in **6b**. The same experimental protocol was applied to functionalized BODIPY **6a**, leading to the desired trivalent cluster **12a** in 83% yield. *O*-Deacetylation of compounds **12** using anion exchange Amberlite IRA-400 (OH^−^) resin provided the desired water-soluble clusters **13** in high yields. As judged by ^11^B NMR, no fluoride displacement occurred at the boron center during the deprotection step.

**Scheme 3:**
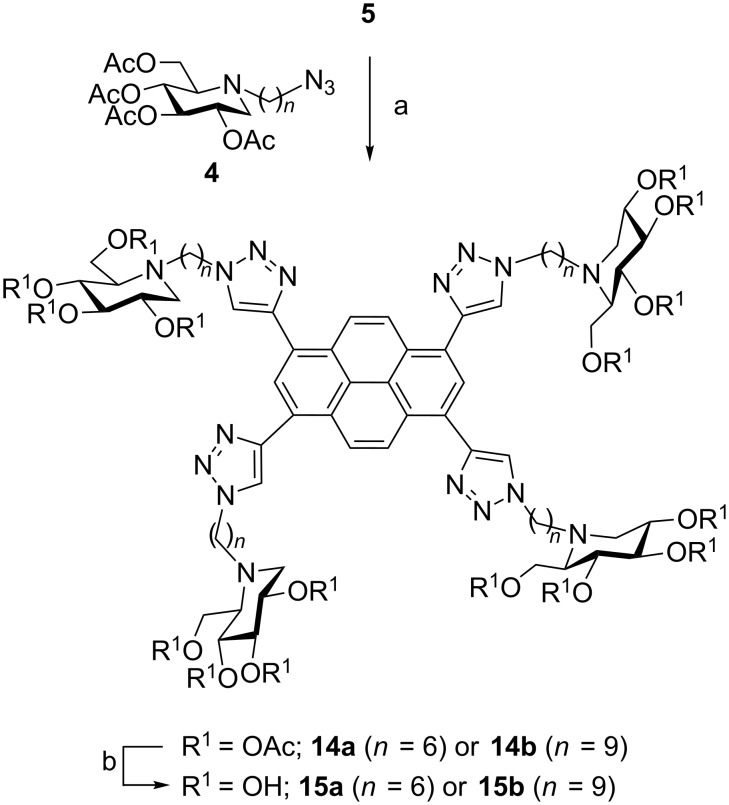
Synthesis of DNJ clusters **15**: (a) CuSO_4_·5H_2_O cat., sodium ascorbate, DMF/H_2_O (6:1), 80 °C (MW) or room temperature, 51% (**14a**), 75% (**14b**); (b) Amberlite IRA 400 (OH^−^), MeOH/H_2_O (1:1), 40 °C, 73% (**15a**), 96% (**15b**).

The synthesis of the 4-valent pyrene-based iminosugars **15** was performed in a similar manner than for BODIPY-based clusters **13** (Scheme 3). The tetrayne **5** synthesized in 3 steps from pyrene [53] was reacted with the azide precursors **4**, and afterwards deprotected to give the desired tetravalent iminosugars **15** in 37 to 72% yields for the two steps. Despite the good water solubility of alkylated DNJ ligands, pyrene-based multivalent iminosugars were only soluble in water/methanol or water/DMSO mixtures, those mixtures prevent the agregation of the pyrene core.

### Photophysical properties

The absorption and emission features of the BODIPY-based cluster **13a** and the pyrene-based cluster **15a** dyes were investigated in an aqueous buffer solution of glycine (0.1 M) at pH 10.7. This buffer conditions were chosen to be as close as possible to the conditions used for β-glucocerebrosidase activation assays (Gaucher disease) which are based on a fluorescent leaving group (4-methylumbelliferone) allowing fluorescence recording after reaction quenching at pH 10.7 [64].

**Figure 3:**
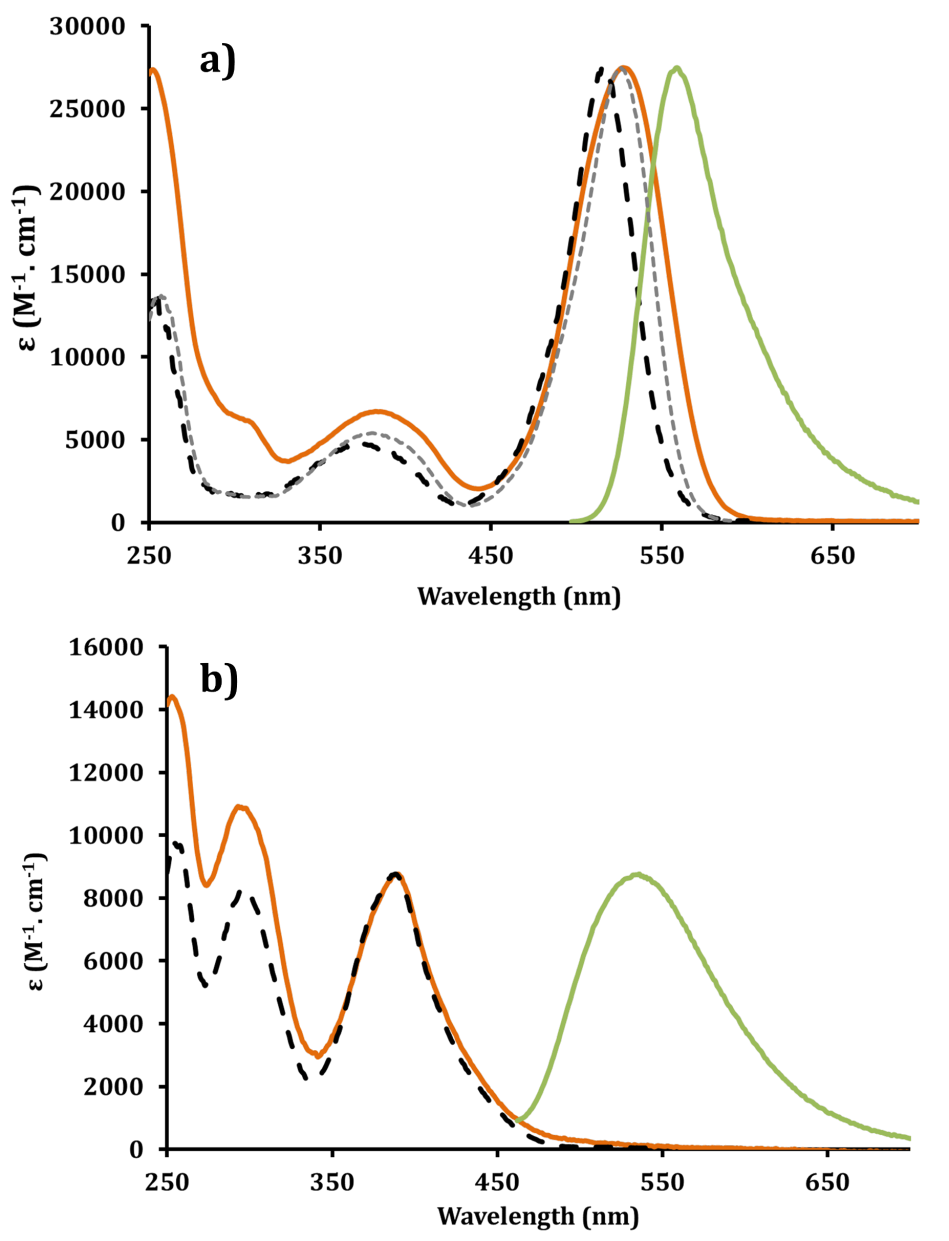
a) Absorption (orange line), corrected emission (green line) (λ_exc_ = 510 nm), excitation (dashed black line) (λ_em_ = 630 nm) and excitation with 2.5% of SDS (small dashed grey line) spectra for **13a**; b) Absorption (orange line), corrected emission (green line) (λ_exc_ = 390 nm) and excitation (dashed black line) (λ_em_ = 550 nm) spectra for **15a**; in glycine buffer at pH 10.7 at rt.

The BODIPY-based dye **13a** displays an intense absorption at 528 nm (ε = 27,000 M^−1^·cm^−1^) corresponding to the S_0_→S_1_ (π–π* transition). The slight red shift of this absorption compared to unsubstituted BODIPY dyes in the 2,6-substitution positions and measured under similar aqueous conditions, is likely due to the influence of both triazole rings. The second transition at 386 nm is assigned in light of previous studies to the S_0_→S_2_ of the BODIPY subunit [39,65–67]. The triazole rings absorb below 250 nm for the π–π* transition [68]. Excitation at 510 nm affords a relatively intense emission with a quantum yield of 24% (in aqueous glycine buffer at pH 10.7), the profile of the band mirrors the absorption with a maximum at 558 nm which is in keeping with little reorganization in the excited state and characteristic of a singlet emitter. The modest Stokes shift (Δ_ss_ = 1020 cm^−1^) and the short excited state life time (τ = 3.38 ns) are also in favor of a singlet emitting state. The excitation spectra did display a slight shift compared to the absorption spectra. This may be due to the presence of some aggregates, a problem frequently encountered with aromatic organic dyes in aqueous solutions [69–70]. Addition of 2.5% of a surfactant such as sodium dodecyl sulfate (SDS) improves the spectral overlap with the absorption spectra (Figure 3a), and likely diminishes formation of potent aggregates.

For the pyrene-based cluster **15a** two main absorptions maxima at 391 and 292 nm were observed and safely assigned to the successive pyrene excited states, S_0_→S_1_ at 391 nm and S_0_→S_2_ at 295 nm (Figure 3b) [71]. Emission maximum was recorded at 534 nm from an excitation at 390 nm or 295 nm with a quantum of 43% (in aqueous glycine buffer at pH 10.7). Unlike the BODIPY homologue, the pyrene-based cluster **15a** has a large Stokes shift of 6850 cm^−1^ and a longer excited state life time (τ = 71.7 ns) [72]. Again the excitation spectrum matches the absorption one proving that aggregation is unlikely under the used aqueous conditions.

From a general point of view, fluorescent probes have been used for the detection of diverse analytes and in relevant biosensing and bioimaging applications [73]. One critical aspect for the evaluation of biological activities using fluorescent dyes (e.g., the deprotonated form of 4-methylumbelliferone) [74] is to determine their spectroscopic features in different environments (local pH, local polarity, potential quenchers, hydrophobic environment, …). Here we focus on the UV–visible characteristics of the anion of 4-methylumbelliferone, the dye commonly used for quantifying chaperoning activities (using 4-methylumbelliferyl β-D-glucopyranoside as GCase substrate) [64], to determine whether this assay would be compatible with the evaluation of fluorescent multivalent clusters **13** and **15** as potential pharmacological chaperones.

**Figure 4:**
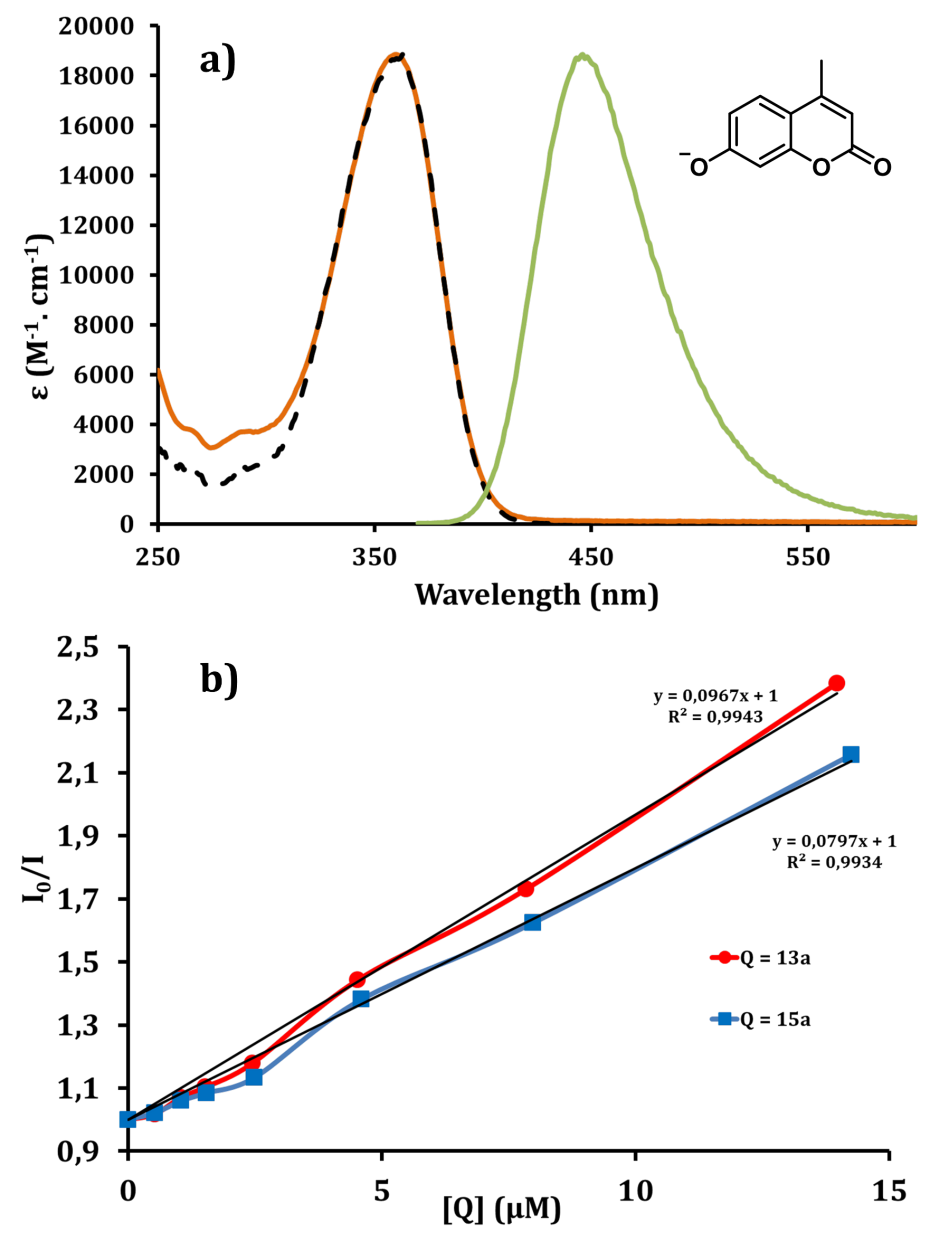
a) Absorption (orange line), corrected emission (green line) (λ_exc_ = 360 nm) and excitation (dashed black line) (λ_em_ = 445 nm) spectra for the anion of 4-methylumbelliferone; b) Stern–Volmer plots concerning the quenching of fluorescence of the anion of 4-methylumbelliferone by **13a** (red line) and **15a** (blue line) and their linear regression.

The same buffer conditions as those used for activation assays (quenched conditions at pH 10.7 in a glycine buffer) were used for this study. The anion of 4-methylumbelliferone displays a strong absorption at 360 nm and a broad emission around 446 nm (Figure 4a).

The fluorescence quantum yield is high (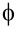
_fluo_ = 81%) as previously determined under similar conditions [74]. In order to record the efficiency of the fluorescence quenching of the anion of 4-methylumbelliferone by the novel dyes, Stern–Volmer plots were carried out [75]. A titrated solution of the quencher (**13a** or **15a**) was dropwise added to a titrated solution of 4-methylumbelliferone (≈10^−7^ M at pH 10.7) and the fluorescence of the anion was recorded after each addition (Figure 4b). This allows plotting the decrease of fluorescence versus the concentration of quencher. The Stern–Volmer equation 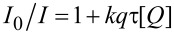 facilitates the calculation of the rates of bimolecular collisional quenching *k*_q_ = 1.8 × 10^13^ M^−1^·s^−1^ and 1.5 × 10^13^ M^−1^·s^−1^, respectively for **13a** and **15a** dyes using a lifetime τ = 5.31 ns for the 4-methylumbelliferone anion. The quenching appears efficient in both cases due to suitable spectral overlap between the emission of 4-methylumbelliferone anion and the absorption of the BODIPY **13a** or the pyrene-based cluster **15a**. This dynamic quenching process between these multivalent iminosugars and the 4-methylumbelliferone or other coumarine derivatives has thus to be taken into account during the quantitative analyses of dedicated biological processes.

## Conclusion

We have reported the preparation of multivalent iminosugar clusters based on two fluorescent cores by way of Cu(I)-catalysed azide–alkyne cycloadditions. To our knowledge these are the first examples of the use of BODIPY or pyrene as a scaffold to display multivalent ligands. Although the trivalent BODIPY-derived DNJ clusters are water soluble, a co-solvent is necessary to dissolve the tetravalent pyrene-derived DNJ clusters in water. Photophysical properties of those multivalent dyes in aqueous media (glycine buffer at pH 10.7), are interesting, providing high quantum yields, 24% for **13a** and 43% for **15a**, and well-defined spectroscopic features. Altogether, these results augur well for a new class of molecular tools dedicated to rationalize the mode of action of pharmacological chaperones and CFTR correctors by probing uptake and mapping biodistribution in cells and in vivo.

## Supporting Information

File 1 Characterization data and NMR spectra of all new compounds.
