# Supplementary material for: Design, synthesis and photochemical properties of the first examples of iminosugar clusters based on fluorescent cores
Source: Beilstein J Org Chem. 2015 May 6;11:659–67. doi: 10.3762/bjoc.11.74 (PMC4464267; doi:10.3762/bjoc.11.74)

Supporting Information

for

Design, synthesis and photochemical properties of the first examples of iminosugar clusters based on fluorescent cores

Mathieu L. Lepage[1], Antoine Mirloup[2], Manon Ripoll[1], Fabien Stauffert[1], Anne Bodlenner[1], Raymond Ziessel*[2] and Philippe Compain*[1,3]

Address: [1]Laboratoire de Synthèse Organique et Molécules Bioactives (SYBIO), Université de Strasbourg/CNRS (UMR 7509), Ecole Européenne de Chimie, Polymères et Matériaux (ECPM), 25 rue Becquerel, 67087 Strasbourg, France, [2]Institut de Chimie et Procédés pour l'Energie, l'Environnement et la Santé (ICPEES), Laboratoire de Chimie Organique et Spectroscopie Avancées (LCOSA), Université de Strasbourg/CNRS (UMR 7515), Ecole Européenne de Chimie, Polymères et Matériaux (ECPM), 25 rue Becquerel, 67087 Strasbourg, France and [3]Institut Universitaire de France, 103 Bd Saint-Michel, 75005 Paris, France

Email: Raymond Ziessel - ziessel@unistra.fr; Philippe Compain - philippe.compain@unistra.fr

*Corresponding author

General information

All the reactions were carried out in standard glassware or in vials adapted to a Biotage Initiator® microwave reactor. Most of the crude mixtures were purified by flash chromatography on silica gel column with silica gel 60 (230-400 mesh, 0.040–0.063 mm) purchased from E. Merck. Automatic flash chromatographies were carried out in a Grace Reveleris® flash system equipped with UV–vis and ELSD detectors. Reaction monitoring and primary characterization of products were achieved by thin layer chromatography (TLC) on aluminum sheets coated with silica gel 60 F254 purchased from E. Merck. Eluted TLC's were revealed under UV (366 nm and 254 nm) and with chemicals (phosphomolybdic acid or potassium permanganate). Nuclear magnetic resonance (NMR) spectra were recorded on a Bruker AC 300, Bruker AC 400 with solvent peaks as reference. ^{1}H and ^{13}C signals were assigned by correlation spectroscopy (COSY), Heteronuclear single quantum correlation (HSQC), and heteronuclear multiple-bond correlation spectroscopy (HMBC). In the following NMR assignments, coupling constants (*J*) will be expressed in Hertz (Hz), multiplicity are described with (s) as singlet, (d) as doublet, (t) as triplet and (q) as quadruplet, “br” prefix means that the considered signal is broad. The indexes “a” or “b” will be used for diastereotopic protons, “a” being assigned to the proton with highest chemical shift and “b” to the proton with the lowest chemical shift. For compounds **13** (12H) and **15** (16H) the hydroxy protons of the sugar are exchanged by residual water or deuterated methanol. Infrared (FTIR) spectra (cm^{-1}) were recorded neat on a Perkin-Elmer Spectrum One Spectrophotometer. ESI–TOF high resolution mass spectra (HRMS) were carried out on a Bruker MicroTOF spectrometer. MALDI–TOF (matrix-assisted laser desorption/Ionisation) mass spectra (MS) were carried out on a Bruker Autoflex II TOF/TOF spectrometer (matrix = dithranol). Elemental analyses were performed with a Thermo Finnigan Flash EA 1112 series. Specific rotations were determined on a Perkin-Elmer 241 polarimeter with sodium lamp (λ = 589 nm). The concentration (*c*) is indicated in decagram per liter (dag/L). Absorption spectra were recorded on a Schimadzu UV-3000 absorption spectrometer. The steady-state fluorescence emission and excitation spectra were obtained by using a HORIBA JOBIN YVON FLUOROMAX 4. All fluorescence spectra were corrected. The fluorescence quantum yield (Φ_{exp}) was calculated from equation 1.

$$\phi_{exp} = \phi_{Ref} \frac{I}{I_{Ref}} \frac{OD_{Ref}}{OD} \frac{\eta^2}{\eta^2{}_{Ref}}$$

(eq. 1)

Here, *I* denotes the integral of the corrected emission spectrum, OD is the optical density at the excitation wavelength and η is the refractive index of the medium. The reference systems

used were rhodamine 6G (Φ_{Fluo} = 0.78) [S1] in air-equilibrated water and tetramethoxydiisoindomethene difluoroborate (Φ_{Fluo} = 0.51) [S2]. Luminescence lifetimes were measured on an Edinburgh Instruments spectrofluorimeter equipped with a R928 photomultiplier and a PicoQuant PDL 800-D pulsed diode connected to a GwInstect GFG-8015G delay generator. No filter was used for the excitation. Emission wavelengths were selected by a monochromator. Lifetimes were deconvoluted with FS-900 software using a light-scattering solution (LUDOX) for instrument response. In the following sections, solvents will be abbreviated as follows: DCM = dichloromethane, AcOEt = ethyl acetate, THF = tetrahydrofuran, MeCN = acetonitrile.

Compound 9:

A solution of **8** (424.0 g, 0.6041 mmol) in THF (20 mL) and triethylamine (10 mL) was placed in a Schlenk tube and degassed with argon for 30 min. Ethynyltrimethylsilane (0.7 mL, 4.8328 mmol, 8 equiv), [Pd(PPh_3)$_2$$Cl_2$] (84.0 mg, 0.1208 mmol, 0.2 equiv) and CuI (46.0 mg, 0.2416 mmol, 0.4 equiv) were then added to the solution. The mixture was heated at 60 °C overnight. Once cooled down to room temperature, the mixture was washed with water. The aqueous phase was extracted with DCM. The combined organic phases were dried over $MgSO_4$ or absorbent cotton and the solvents were evaporated under reduced pressure. The crude product was purified by flash chromatography (Petroleum ether/DCM 80:20) to afford **9** (326.7 mg, 0.533 mmol, 88%) as a red solid. FTIR (neat) ν_{max}, 2153 (C≡C), 1531 (C=C), 1475, 1365, 1393, 1249, 1317, 1199, 1175, 1090, 1006, 837, 758, 706, 588 cm^{-1}; ^{1}H-NMR ($CDCl_3$, 300 MHz) δ 7.61 (d, J = 7.8 Hz, 2H; H-1), 7.19 (d, J = 7.9 Hz, 2H; H-2), 2.63 (s, 6H; H-3), 1.47 (s, 6H; H-4), 0.29 (s, 9H; H-5), 0.21 ppm (s, 18H; H-6); ^{13}C-NMR ($CDCl_3$, 75 MHz) δ 158.2, 143.8, 140.9, 133.6, 132.0, 129.9, 127.0, 123.5, 115.6, 103.1, 101.1, 96.1, 95.4, 12.7, -0.8, -1.0 ppm; MS (ESI) m/z : 613.1 ($[M + H]^+$); Anal. Calcd for $C_{34}H_{43}BF_2N_2Si_3$: C, 66.64; H, 7.07; N, 4.57; found: C, 66.43; H, 6.83; N, 4.35.

Compound 6a:

K_2CO_3 (1.4 g, 10.1078 mmol, 20 equiv) was added to a solution of **9** (309.7 mg, 0.5054 mmol) in DCM (10 mL), MeOH (5 mL) and water (5 mL). The solution was heated at 50 °C overnight. Once cooled down to room temperature, the mixture was washed with water. The aqueous phase was extracted with DCM. The combined organic phases were dried over $MgSO_4$ or absorbent cotton and the solvents were evaporated under reduced pressure. The crude product was purified by flash chromatography (petroleum ether/DCM 70:30) to afford

6a (145.0 mg, 0.3659 mmol, 72%) as a red solid. FTIR (neat) ν_{max} 3267 (≡C-H), , 2101 (C≡C), 1531 (C=C), 1471, 1401, 1314, 1114, 1003, 840, 767, 680, 583 cm^{-1}; ^{1}H-NMR ($CDCl_3$, 400 MHz) δ 7.66 (d, J = 8.1 Hz, 2H; H-1), 7.24 (d, J = 8.1 Hz, 2H; H-2), 3.32 (s, 2H; H-6), 3.21 (s, 1H; H-5), 2.65 (s, 6H; H-3), 1.49 ppm (s, 6H; H-4); ^{13}C-NMR ($CDCl_3$, 100 MHz) δ 158.3, 144.4, 141.1, 133.9, 132.2, 129.8, 127.1, 122.7, 114.5, 83.5, 81.8, 78.2, 74.9, 12.7, 12.6 ppm; MS (ESI) m/z : 397.1 ($[M + H]^+$); Anal. Calcd for $C_{25}H_{19}BF_2N_2$: C, 75.78; H, 4.83; N, 7.07; found: C, 75.54; H, 4.62; N, 6.87.

Compound 10:

In a similar manner as described in reference [S3], in a Schlenk tube was added ethylmagnesium bromide (3.2 mL, 2.883 mmol, 5 equiv) to a stirred solution of 2,5,8,11,14-pentaoxaheptadec-16-yne (852.2 mg, 3.460 mmol, 6 equiv) in anhydrous THF. The mixture was stirred at 60 °C for 2 h. The resulting solution was then transferred via cannula to a solution of **8** (404.8 mg, 0.5767 mmol) in anhydrous THF. The solution was stirred at 60 °C overnight. Once cooled down at room temperature, the mixture was washed with 1 M HCl and water. The aqueous phase was extracted with DCM. The combined organic phases were dried over $MgSO_4$ or absorbent cotton and the solvents were evaporated under reduced pressure. The resulting crude residue was purified by flash chromatography (AcOEt/MeOH 90:10) to afford **10** (408.2 mg, 0.3354 mmol, 61%) as a red oil. FTIR (neat) ν_{max}, 1531 (C=C), 1341, 1304, 1160, 1092, 994 , 970, 849, 748, 694, 587, 533 cm^{-1}; ^{1}H-NMR ($CDCl_3$, 400 MHz) δ 7.78 (d, J = 8.2 Hz, 2H; H-1), 6.96 (d, J = 8.2 Hz, 2H; H-2), 4.08 (s, 4H; H-5), 3.52-3.59 (m, 28H; H-6 to H-12), 3.42-3.47 (m, 4H; H-13), 3.27 (s, 6H; H-14), 2.73 (s, 6H; H-3), 1.33 ppm (s, 6H; H-4); ^{13}C-NMR ($CDCl_3$, 100 MHz) δ 156.2, 142.9, 139.3, 138.3, 134.5, 129.7, 128.9, 95.0, 91.3, 86.2, 71.7, 70.4, 70.3, 70.3, 70.3, 70.2, 68.7, 59.3, 58.8, 17.5, 17.3 ppm; MS (ESI) m/z : 1155.1 ($[M + H]^+$); Anal. Calcd for $C_{43}H_{58}BI_3N_2O_{10}$: C, 44.74; H, 5.06; N, 2.43; found: C, 44.52; H, 4.62; N, 2.13.

Compound 11:

A solution of **10** (327.4 g, 0.2836 mmol) in THF (5 mL) and triethylamine (5 mL) was placed in a Schlenk tube and degassed with argon for 30 min. Ethynyltrimethylsilane (0.3 mL, 2.2688 mmol, 8 equiv), $[Pd(PPh_3)_2Cl_2]$ (40.0 mg, 0.05672 mmol, 0.2 equiv) and CuI (21.0 mg, 0.1134 mmol, 0.4 equiv) were then added to the solution. The mixture was heated at 60 °C overnight. Once cooled down to room temperature, the mixture was washed with water. The

aqueous phase was extracted with DCM. The combined organic phases were dried over $MgSO_4$ or absorbent cotton and the solvents were evaporated under reduced pressure. The crude product was purified by flash chromatography (AcOEt/MeOH 95:5) to afford **11** (287.5 mg, 0.2699 mmol, 95%) as a red oil. FTIR (neat) ν_{max}, 2148 (C≡C), 1535 (C=C), 1315, 1248, 1190, 1099, 1014, 836, 759, 706, 595 cm^{-1}; ^1H-NMR ($CDCl_3$, 400 MHz) δ 7.60 (d, J = 8.3 Hz, 2H; H-1), 7.23 (d, J = 8.2 Hz, 2H; H-2), 4.16 (s, 4H; H-5), 3.62-3.66 (m, 28H; H-6 to H-12), 3.52-3.55 (m, 4H; H-13), 3.37 (s, 6H; H-14), 2.79 (s, 6H; H-3), 1.45 (s, 6H; H-4), 0.28 (s, 9H; H-15), 0.20 ppm (s, 18H; H-16); ^{13}C-NMR ($CDCl_3$, 100 MHz) δ 157.7, 142.0, 140.7, 134.2, 131.9, 128.3, 127.2, 123.3, 115.6, 103.3, 100.9, 96.8, 95.1, 90.5, 71.1, 69.7, 69.7, 69.7, 69.7, 69.6, 68.0, 58.6, 58.2, 14.2, 12.9, -0.7, -1.0 ppm; MS (ESI) m/z : 1065.2 ($[M + H]^+$), 991.3; Anal. Calcd for $C_{58}H_{85}BN_2O_{10}Si_3$: C, 65.39; H, 8.04; N, 2.63; found: C, 65.22; H, 7.73; N, 2.38.

Compound 6b:

K_2CO_3 (1.8 g, 12.8078 mmol, 50 equiv) was added to a solution of **11** (272.9 mg, 0.2562 mmol) in DCM (10 mL), MeOH (5 mL) and water (5 mL). The solution was stirred at room temperature overnight. The mixture was then washed with water. The aqueous phase was extracted with DCM. The combined organic phases were dried over $MgSO_4$ or absorbent cotton and the solvents were evaporated under reduced pressure. The crude product was purified by flash chromatography (AcOEt/MeOH 95:5) to afford **6b** (190.7 mg, 0.2247 mmol, 88%) as a red oil. FTIR (neat) ν_{max} 3233 (≡C-H), , 2104 (C≡C), 1533 (C=C), 1471, 1392, 1365, 1312, 1179, 1092, 1064, 1012, 840, 707, 591 cm^{-1}; ^1H-NMR ($CDCl_3$, 400 MHz) δ 7.63 (d, J = 8.2 Hz, 2H; H-1), 7.25 (d, J = 8.2 Hz, 2H; H-2), 4.16 (s, 4H; H-5), 3.58-3.65 (m, 28H; H-6 to H-12), 3.50-3.54 (m, 4H; H-13), 3.35 (s, 6H; H-14), 3.33 (s, 2H; H-16), 3.20 (s, 1H; H-15), 2.79 (s, 6H; H-3), 1.46 ppm (s, 6H; H-4); ^{13}C-NMR ($CDCl_3$, 100 MHz): δ = 158.8, 143.5, 141.8, 135.3, 133.1, 129.1, 128.3, 123.4, 115.5, 91.6, 84.4, 82.8, 78.9, 76.4, 72.0, 70.7, 70.6, 70.6, 70.5, 69.0, 59.6, 59.1, 15.1, 13.7 ppm; MS (ESI) m/z : 849.4 ($[M + H]^+$); Anal. Calcd for $C_{49}H_{61}BN_2O_{10}$: C, 69.33; H, 7.24; N, 3.30; found: C, 69.04; H, 7.02; N, 3.09.

General procedure for the synthesis of iminosugar click clusters 12:

In a similar manner as described in reference [16], to a solution of **6a** or **6b** and azide-armed DNJ ligand **4a** (1.5 equiv/alkyne moiety) in THF under argon (solution degassed by bubbling 5 min with argon) was added a bright yellow solution of $CuSO_4{\cdot}5H_2O$ (15 to 20 mol %/alkyne moiety) and sodium ascorbate (30 to 40 mol %/alkyne moiety) in H_2O (solution degassed by bubbling 5 min with argon) at room temperature. The colored mixture darkened rapidly and

was further stirred for 16 to 20 h at room temperature. The mixture was diluted with a 2:2:1 mixture of MeCN/AcOEt/30% NH_4OH and filtered through a pad of silica gel, using the same mixture as eluent (25 mL). Copper salts precipitated as a blue powder and remained at the top of the silica gel pad. The filtrate was concentrated and the crude residue was purified by flash chromatography (DCM/MeOH 100:0 to 95:5) to afford iminosugar click clusters **12a** and **b** as red solids.

General procedure for the synthesis of iminosugar click clusters 14:

In a similar manner as described in reference [16], to a solution of the 1,3,6,8-tetraethynylpyrene **5** and azide-armed DNJ ligand **4a**-**b** (1.25 to 1.5 equiv/alkyne moiety) in DMF was added a bright yellow suspension of $CuSO_4{\cdot}5H_2O$ (10 mol %/alkyne moiety) and sodium ascorbate (20 mol %/alkyne moiety) in water. The reaction mixture was stirred and heated under microwave irradiation for 50 min at 80°C for compound **14a** or stirred at room temperature for 3 days for compound **14b**. Then the mixture was concentrated under reduced pressure, diluted in a 9:1:1 (v/v/v) mixture of MeCN/H_2O/30% NH_4OH and filtered with the same eluent (25 mL) on a small pad of silica gel. Copper salts precipitated as a blue powder and remained at the top of the silica gel pad. The filtrate was concentrated and the crude residue was purified by flash chromatography (AcOEt/petroleum ether 40:60 then DCM/MeOH 15:1) to afford iminosugar click clusters **14a** and **b** as yellow oils.

General procedure for the synthesis of deprotected iminosugar click clusters 13 and 15:

In a similar manner as described in reference [16], to a solution of acetylated iminosugar click clusters **12a**,**b** or **14a**,**b** in a 1:1 mixture of H_2O/MeOH was added Amberlite IRA400 (OH^-) (5.5*n* g/mmol of substrate; *n* = number of acetate groups). The suspension was gently stirred overnight at 40 °C or room temperature. Then the mixture was filtered and the resin was rinsed with a 1:1 mixture of H_2O/MeOH. The filtrate was concentrated to afford deprotected iminosugar click clusters **13a**,**b**, **15a**,**b** in quantitative yields.

Compound 12a:

$[\alpha]_D^{20} = +10$ (*c* 0.1, $CHCl_3$); m.p. 210.0-211.5 °C; FTIR (neat) ν_{max} 1743 cm^{-1}; ^1H-NMR ($CDCl_3$, 400 MHz) δ 8.01 (d, J = 8.2 Hz, 2H; H-7' or H-8'), 7.88 (s, 1H; H-11m), 7.46 (s, 2H;

H-11β), 7.40 (d, J = 8.2 Hz, 2H; H-7'or H-8'), 5.11-4.99 (m, 6H; H-3, H-4), 4.99-4.88 (m, 3H; H-2), 4.43 (t, J = 7.3 Hz, 2H; H-12m), 4.38 (t, J = 7.3 Hz, 4H; H-12β), 4.24-4.06 (m, 6H; H-6), 3.24-3.13 (m, 3H; H-1a), 2.81-2.65 (m, 3H; H-7a), 2.69 (s, 6H; H-13'), 2.65-2.56 (m, 3H; H-5), 2.56-2.44 (m, 3H; H-7b), 2.32-2.22 (m, 3H; H-1b), 2.10-1.98 (several singlets, 36H; Ac), 2.01-1.88 (m, 6H; H-11), 1.53 (s, 6H; H-12'), 1.50-1.22 ppm (m, 18H; H-8, H-9, H-10); ^{13}C-NMR ($CDCl_3$, 100 MHz) δ 171.0, 170.5, 170.22, 170.19, 169.9, 155.5, 147.0, 140.6, 140.5, 134.8, 131.9, 131.5, 128.8, 126.6, 122.8, 122.1, 120.1, 74.7, 69.6, 69.5, 61.9, 61.8, 59.6, 52.9, 51.6, 50., 50.3, 30.3, 26.7, 26.5, 25.0, 21.01, 21.99, 20.95, 20.9, 20.8, 13.9, 13.4 ppm; HRMS (ESI) m/z calcd for $C_{85}H_{117}BF_2N_{14}O_{24}$ $[M + 2H]^{2+}$ 883.4208; found 883.4228.

Compound 13a:

Optical rotation could not be measured because of intense red color of **13a**; m.p. 167.8-169.8 °C; FTIR (neat) ν_{max} 3370, 1590 cm^{-1}; ^{1}H-NMR (CD_3OD, 400 MHz) δ 8.48 (s, 1H; H-11m), 8.07 (d, J = 8.1 Hz, 2H; H-8'), 8.05 (s, 2H; H-11β), 7.49 (d, J = 8.1 Hz, 2H; H-7'), 4.48 (t, J = 7.0 Hz, 2H; H-12m), 4.44 (t, J = 7.0 Hz, 4H; H-12β), 3.77-3.87 (m, 6H; H-6), 3.41-3.49 (m, 3H; H-2), 3.32-3.35 (m, 3H; H-4), 3.11 (t, J = 9.0 Hz, 3H; H-3), 2.93-2.99 (m, 3H; H-1a), 2.71-2.82 (m, 3H; H-7a), 2.61 (br s, 6H; H-13'), 2.48-2.58 (m, 3H; H-7b), 2.10-2.18 (m, 3H; H-1b), 2.04-2.10 (m, 3H; H-5), 1.90-2.03 (m, 6H; H-11), 1.53 (br s, 6H; H-12'), 1.43-1.52 (m, 6H; H-8), 1.28-1.41 ppm (m, 12H; H-9, H-10); ^{13}C-NMR (CD_3OD, 100 MHz) δ 156.5, 147.9, 144.3, 142.0, 141.0, 135.9, 133.2, 132.6, 130.2, 127.7, 125.1, 123.9, 122.9, 80.6, 72.1, 70.8, 67.5, 59.6, 57.7, 53.6, 51.5, 51.4, 31.2, 27.9, 27.4, 25.2, 13.9, 13.6 ppm; ^{19}F-NMR (CD_3OD/D_2O 1:1, 376.5 MHz) δ -141.4 ppm; ^{11}B-NMR (CD_3OD/D_2O 1:1, 128 MHz) δ 2.05 ppm (t, J = 32.8 Hz, BF_2); HRMS (ESI) m/z calcd for $C_{61}H_{93}BF_2N_{14}O_{12}$ $[M + 2H]^{2+}$ 631.3574; found 631.3505.

Compound 14a:

$[\alpha]_D^{20}$ = +2 (c 1, $CHCl_3$); FTIR (neat) ν_{max} 2940, 1743 cm^{-1}; ^{1}H-NMR ($CDCl_3$, 400 MHz) δ 8.80 (s, 4H; H-4', H-5', H-9', H-10'), 8.60 (s, 2H; H-2', H-7'), 8.07 (s, 4H; H-13), 5.10-4.99 (m, 8H; H-3, H-4), 4.99-4.91 (m, 4H; H-2), 4.54 (t, J = 7.0 Hz, 8H; H-12), 4.21-4.10 (m, 8H; H-6), 3.20 (dd, 4H, J = 11.4 Hz, 4.9 Hz; H-1a), 2.82-2.72 (m, 4H; H-7a), 2.67-2.60 (m, 4H; H-5), 2.60-2.51 (m, 4H; H-7b), 2.31 (t, J = 10.9 Hz, 4H; H-1b), 2.09-2.03 (m, 20H; Ac, H-11), 2.01 (s, 12H; Ac) , 2.00 (s, 12H; Ac), 1.99 (s, 12H; Ac), 1.55-1.30 ppm (m, 24 H; H-8, H-9, H-10); ^{13}C-NMR ($CDCl_3$, 100 MHz) δ 171.0, 170.4, 170.2, 169.9, 147.2, 128.8, 128.7, 126.5, 126.2, 125.9, 123.3, 74.8, 69.6, 69.5, 61.8, 59.7, 53.0, 51.7, 50.6, 30.5, 26.8, 26.6, 25.0, 21.0, 20.88, 20.82 ppm; HRMS (ESI) m/z calcd for $C_{104}H_{140}N_{16}O_{32}$ $[M + H]^+$ 2123.9736; found 2123.9852.

Compound 15a:

$[\alpha]_D^{20} = +2$ (c 1, MeOH/$CDCl_3$/H_2O 4:1:1); FTIR (neat) ν_{max} 3290 cm^{-1}; ^{1}H-NMR (MeOD/$CDCl_3$/D_2O 4:1:1, 400 MHz) δ 8.45 (s, 4H; H-4', H-5', H-9', H-10'), 8.38 (s, 2H; H-2', H-7'), 8.33 (s, 4H; H-13), 4.55 (m, 8H; H-12), 3.84 (s, 8H; H-6), 3.51 (ddd, J = 10.4 Hz, 9.4 Hz, 4.9 Hz, 4H; H-2), 3.38 (t, J = 9.4 Hz, 4H; H-4), 3.19 (t, J = 9.2 Hz, 4H; H-3), 3.00 (dd, J = 11.1 Hz, 4.8 Hz, 4H; H-1a), 2.83-2.71 (m, 4H; H-7a), 2.56-2.67 (m, 4H; H-7b), 2.28-2.12 (m, 8H; H-1b, H-5), 2.10-1.97 (m, 8H; H-11), 1.59-1.30 ppm (m, 24 H; H-8, H-9, H-10); ^{13}C-NMR (MeOD/$CDCl_3$/D_2O 4:1:1, 100 MHz) δ 147.2, 129.7, 129.1, 129.0, 126.2, 126.1, 125.3, 79.8, 71.3, 70.1, 66.4, 58.8, 57.0, 53.3, 51.5, 31.0, 27.7, 27.2, 24.3, 21.7 ppm; MS (MALDI-TOF) m/z calcd for $C_{72}H_{106}N_{16}NaO_{16}$ $[M + Na]^+$ 1473.786; found 1473.584.

Compound 12b:

$[\alpha]_D^{20} = -9$ (c 0.1, $CHCl_3$); FTIR (neat) ν_{max} 1745 cm^{-1}; ^{1}H-NMR ($CDCl_3$, 400 MHz) δ 7.99 (d, J = 8.2 Hz, 2H; H-7' or H-8'), 7.87 (s, 1H; H-11m), 7.46 (s, 2H; H-11β), 7.42 (d, J = 8.2 Hz, 2H; H-7' or H-8'), 5.11-4.98 (m, 6H; H-3, H-4), 4.98-4.88 (m, 3H; H-2), 4.42 (t, J = 7.2 Hz, 2H; H-12m), 4.37 (t, J = 7.2 Hz, 4H; H-12β), 4.18 (s, 4H; H-16'), 4.21-4.05 (m, 6H; H-6), 3.72-3.47 (m, 32H; H-17' to H-24'), 3.36 (s, 6H; H-25'), 3.18 (m, 3H; H-1a), 2.84 (s, 6H; H-13'), 2.73 (m, 3H; H-7a), 2.61 (m, 3H; H-5), 2.51 (m, 3H; H-7b), 2.27 (t, J =11.0 Hz, 3H; H-1b), 2.10-1.98 (several singlets, 36H; Ac), 1.95 (m, 6H; H-11), 1.49 (s, 6H; H-12'), 1.52-1.20 ppm (m, 18H; H-8 to H-10); ^{13}C-NMR ($CDCl_3$, 100 MHz) δ 170.98, 170.96, 170.5, 170.23, 170.2, 169.7, 155.2, 147.2, 142.2, 141.0, 138.7, 135.4, 131.6, 129.8, 129.0, 126.5, 122.9, 122.3, 120.1, 94.1, 91.3, 74.7, 72.1, 70.71, 70.67, 71.65, 70.62, 70.58, 70.57, 69.7, 69.5, 69.0, 61.8, 59.72, 59.66, 59.1, 53.0, 51.6, 50.5, 50.3, 30.5, 26.7, 26.6, 25.1, 21.3-20.3, 15.5, 13.5 ppm; HRMS (ESI) m/z calcd for $C_{109}H_{157}BN_{14}O_{34}Na$ $[M + Na]^+$ 2240.0972; found 2240.1001.

Compound 13b:

$[\alpha]_D^{20} = -19$ (c 0.1, MeOH); FTIR (neat) ν_{max} 3367, 1535 cm^{-1}; ^{1}H-NMR (CD_3OD, 400 MHz) δ 8.48 (s, 1H; H-11m), 8.07 (d, J = 8.2 Hz, 2H; H-8'), 8.04 (s, 1H; H-11β), 7.48 (d, J = 8.2 Hz, 2H; H-7'), 4.49 (t, J = 7.0 Hz, 2H; H-12m), 4.43 (t, J = 7.0 Hz, 4H; H-12β), 4.18 (s, 4H; H-16'), 3.77-3.88 (m, 6H; H-6), 3.49-3.67 (m, 32H; H-17' to H-24'), 3.42-3.48 (m, 3H; H-2), 3.32-3.36 (m, 9H; H-4, H-25'), 3.08-3.14 (m, 3H; H-3), 2.93-2.99 (m, 3H; H-1a), 2.73-2.86 (m, 9H; H-7a, H-13'), 2.49-2.58 (m, 3H; H-7b), 2.05-2.17 (m, 6H; H-1b, H-5), 1.91-2.02 (m, 6H; H-11), 1.29-1.53 ppm (m, 24H; H-8, H-9, H-10, H-12'); ^{13}C-NMR (CD_3OD, 100 MHz) δ 156.3 (C-1'), 147.9

(C-10m), 144.2 (C-6'), 141.3 (C-10β), 140.0 (C3'), 136.4 (C-9'), 133.1 (C-5'), 130.9 (C-4'), 130.3 (C-7'), 127.6 (C-8'), 125.2 (C-11β), 124.0 (C-2'), 122.9 (C-11m), 96.4 (C-14'), 92.6 (C-15'), 80.6 (C-3), 72.9 (C24'), 72.1 (C-4), 71.5, 71.41, 71.35, 71.3 (C-18' to C-23'), 70.8 (C-2), 69.9 (C-17'), 67.5 (C-5), 60.1 (C-16'), 59.6 (C-6), 59.1 (C-25'), 57.7 (C-1), 53.6 (C-7), 51.5 (C-12m), 51.4 (C-12β), 31.2 (C-11), 27.9, 27.4, 25.2 (C-8, C-9, C-10), 15.8 (C13'), 13.7 ppm (C-12'); HRMS (ESI) m/z calcd for $C_{85}H_{129}D_4BN_{14}O_{22}$ $[M + 2H]^{2+}$ 859.5142; found 859.5093.

Compound 14b:

$[\alpha]_D^{21} = +3$ (c 1, $CHCl_3$); FTIR (neat) ν_{max} 2929, 1741 cm^{-1}; ^{1}H-NMR ($CDCl_3$, 400 MHz) δ 8.80 (s, 4H; H-4', H-5', H-9', H-10'), 8.60 (s, 2H; H-2', H-7'), 8,06 (s, 4H; H-16), 5.11-4.92 (m, 12H; H-2, H-3, H-4), 4.54 (t, J = 7.3 Hz, 8H; H-15), 4.15 (m, 8H; H-6), 3.23-3.14 (m, 4H; H-1a), 2.75-2.50 (m, 12H; H-7a, H-5, H-7b), 2.34 (t, J = 9.5 Hz, 4H; H-1b), 2.09-2.03 (m, 20H; H-14, Ac), 2.003 (s, 12H; Ac), 1.995 (s, 24H; Ac), 1.51-1.16 ppm (m, 48 H; H-8 to H-13); ^{13}C-NMR ($CDCl_3$, 100 MHz) δ 171.0, 170.5, 170.1, 169.8, 147.2, 132.2, 128.8, 128.6, 126.2, 125.9, 123.3, 74.8, 69.7, 69.5, 61.7, 59.6, 53.0, 52.0, 50.7, 30.6, 29.5, 29.1, 27.3, 26.8, 24.9, 21.0, 20.9, 20.8 ppm; HRMS (ESI) m/z calcd for $C_{116}H_{164}N_{16}O_{32}$ $[M + H]^+$ 1146.5843; found 1146.5851.

Compound 15b:

$[\alpha]_D^{20} = -44$ (c 0.1, $MeOH/CHCl_3/H_2O$ 4:1:1); FTIR (neat) ν_{max} 3293 cm^{-1}; ^{1}H-NMR ($CD_3OD/CDCl_3/D_2O$ 4:1:1, 400 MHz) δ 8.50 (s, 4H; H-4', H-5', H-9', H-10'), 8.40 (s, 2H; H-2', H-7'), 8.34 (s, 4H; H-16), 4.56 (m, 8H; H-15), 3.84-3.80 (m, 8H; H-6), 3.53-3.45 (m, 4H; H-2), 3.37 (t, J = 9.2 Hz, 4H; H-4), 3.16 (t, J = 9.0 Hz, 4H; H-3), 2.97 (dd, J = 11.2 Hz, 4.8 Hz, 4H; H-1a), 2.79-2.67 (m, 4H; H-7a), 2.65-2.54 (m, 4H; H-7b), 2.22 (t, J = 11.0 Hz, 4H; H-1b), 2.16-2.11 (m, 4H; H-5), 2.10-1.99 (m, 8H; H-14), 1.51-1.10 ppm (m, 48 H; H-8 to H-13); ^{13}C-NMR ($CDCl_3$, 100 MHz) δ 147.3, 129.8, 129.7, 129.6, 129.3, 126.4, 126.3, 125.3, 79.8, 71.3, 70.1, 66.4, 58.8, 57.1, 53.4, 51.6, 33.2, 31.2, 30.3, 29.9, 28.3, 27.4, 26.6, 24.5 ppm; MS (MALDI-TOF) m/z calcd for $C_{84}H_{131}N_{16}O_{16}$ $[M + H]^+$ 1619.993; found 1619.99.

Stern–Volmer plots:

Three stock solutions were prepared.

Solution A: 2.36 mg of 4-methylumbelliferone were diluted in 25 mL of an aqueous buffer solution of glycine (0.1 M) at pH 10.7. This solution was then diluted 1,000 times to give a concentration of $5.36{\cdot}10^{-7}$ M.

Solution B: 0.92 mg of **13a** were diluted in 25 mL of the same buffer solution to give a concentration of $2.92{\cdot}10^{-5}$ M.

Solution C: 0.86 mg of **15a** were diluted in 20 mL of the same buffer solution to give a concentration of $2.96{\cdot}10^{-5}$ M.

Quenching experiments: 2 mL of solution A were placed in a quartz cell. In each case, addition of 36 µL of solution B or C corresponding to one equivalent of **13a** or **15a** in the solution A. Intensity of fluorescence at 446 nm (maximum of emission of the anion of 4-methylumbelliferone) was recorded after each addition (0, 1, 2, 3, 5, 10, 20 and 50 equivalents) of solution B or C directly in the quartz cell. This led to a decrease of fluorescence that is plotted versus the concentration of the quencher (**13a** or **15a**). According to the respective absorption spectrum the excitation wavelength is 340 nm.

References

S1 Olmsted, J., III. *J. Phys. Chem.* **1979,** *83,* 2581–2584. doi:10.1021/j100483a006

S2 Ulrich, G.; Goeb, S.; De Nicola, A.; Retailleau, P.; Ziessel, R. *Synlett* **2007,** *10,* 1517–1520. doi:10.1055/s-2007-982557

S3 Ulrich, G.; Haefele, A.; Retailleau, P.; Ziessel, R. *J. Org. Chem.* **2012,** *77,* 5036–5048. doi:10.1021/jo300477p

Copies of the ^{1}H-NMR and ^{13}C-NMR spectra of 6, 9-15:

^{1}H-NMR spectrum ($CDCl_3$, 300 MHz) of compound 9:

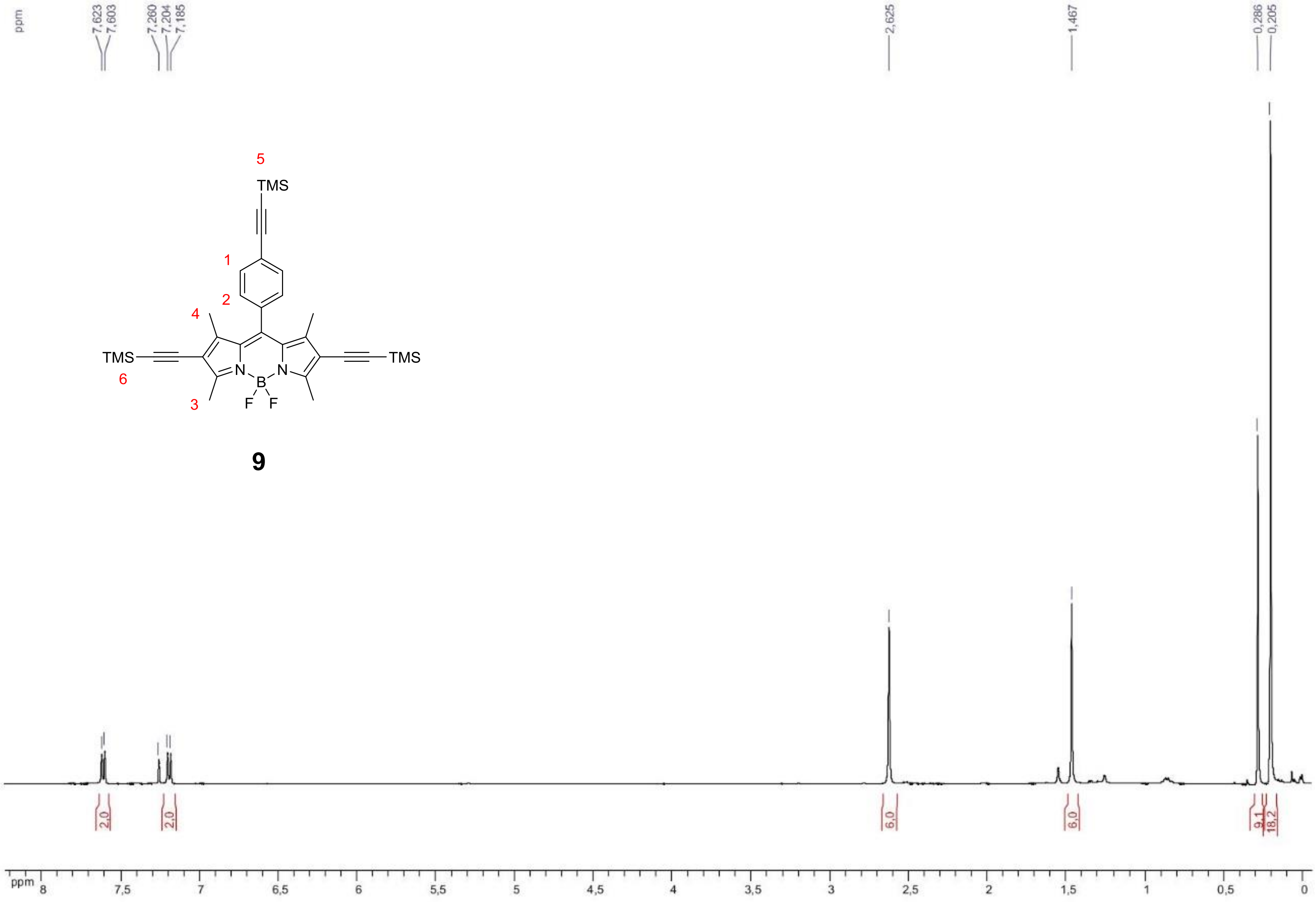

^{13}C-NMR spectrum ($CDCl_3$, 75 MHz) of compound 9:

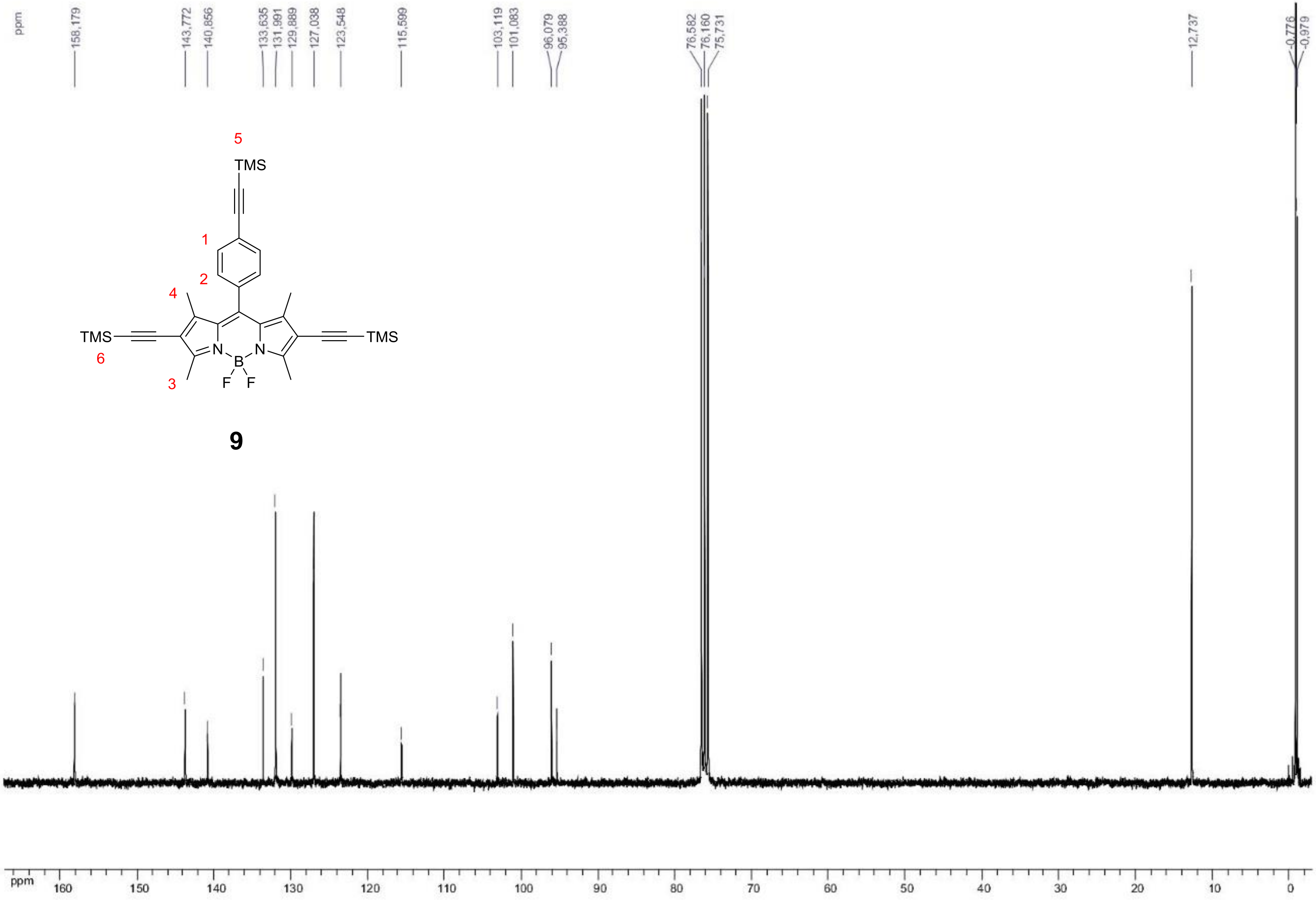

^{1}H-NMR spectrum ($CDCl_3$, 400 MHz) of compound 6a:

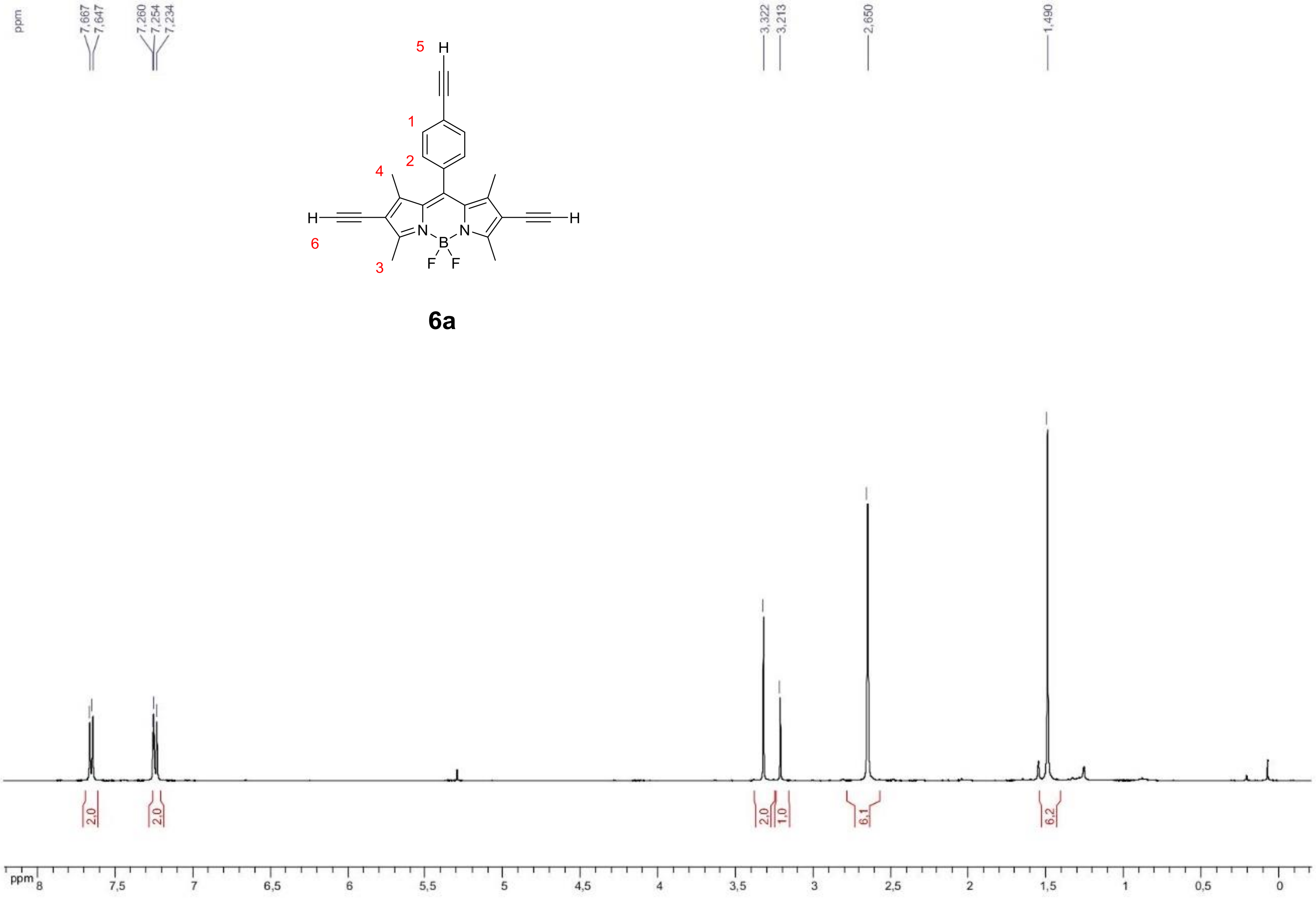

^{13}C-NMR spectrum ($CDCl_3$, 100 MHz) of compound 6a:

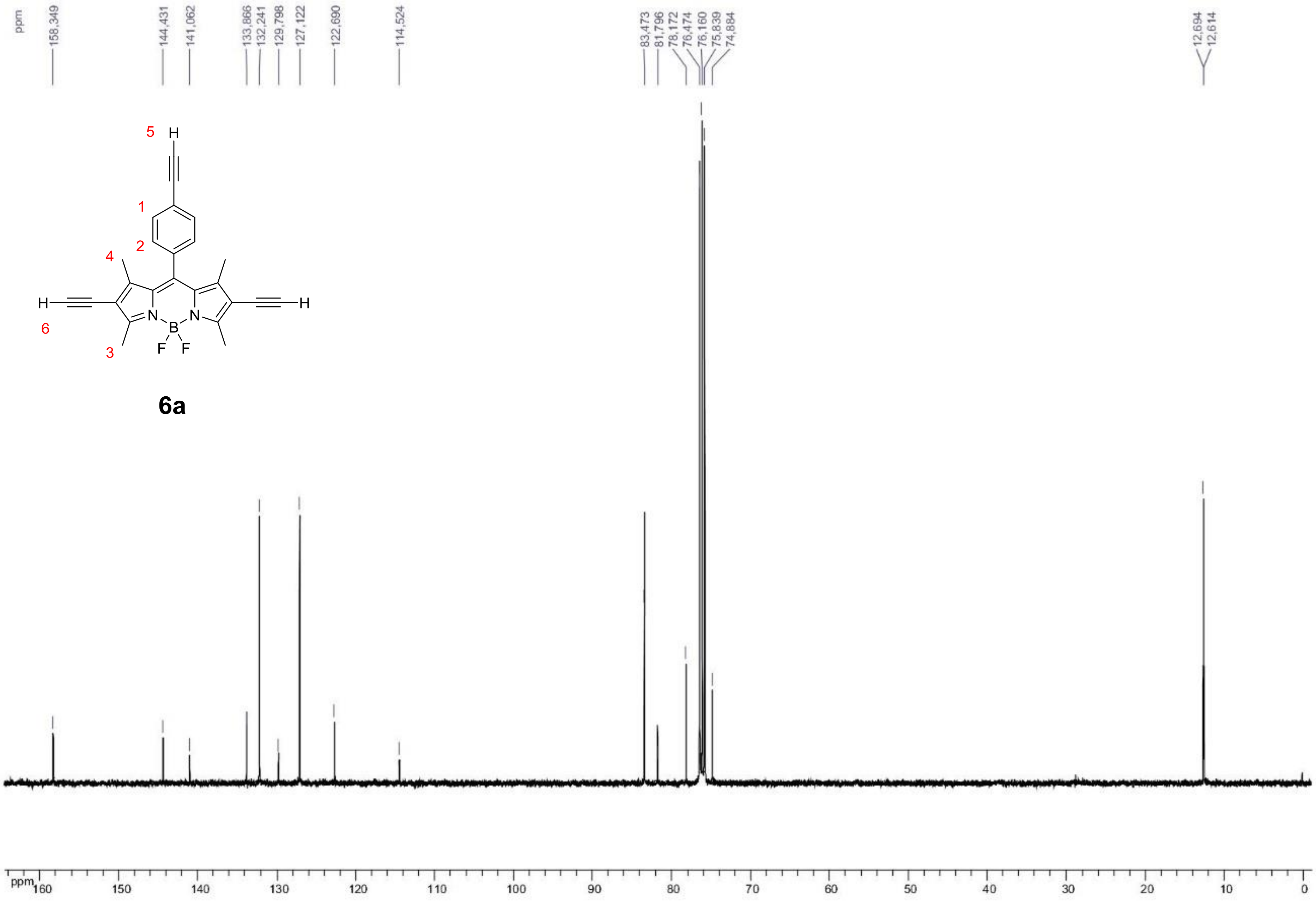

^{1}H-NMR spectrum ($CDCl_3$, 400 MHz) of compound 10:

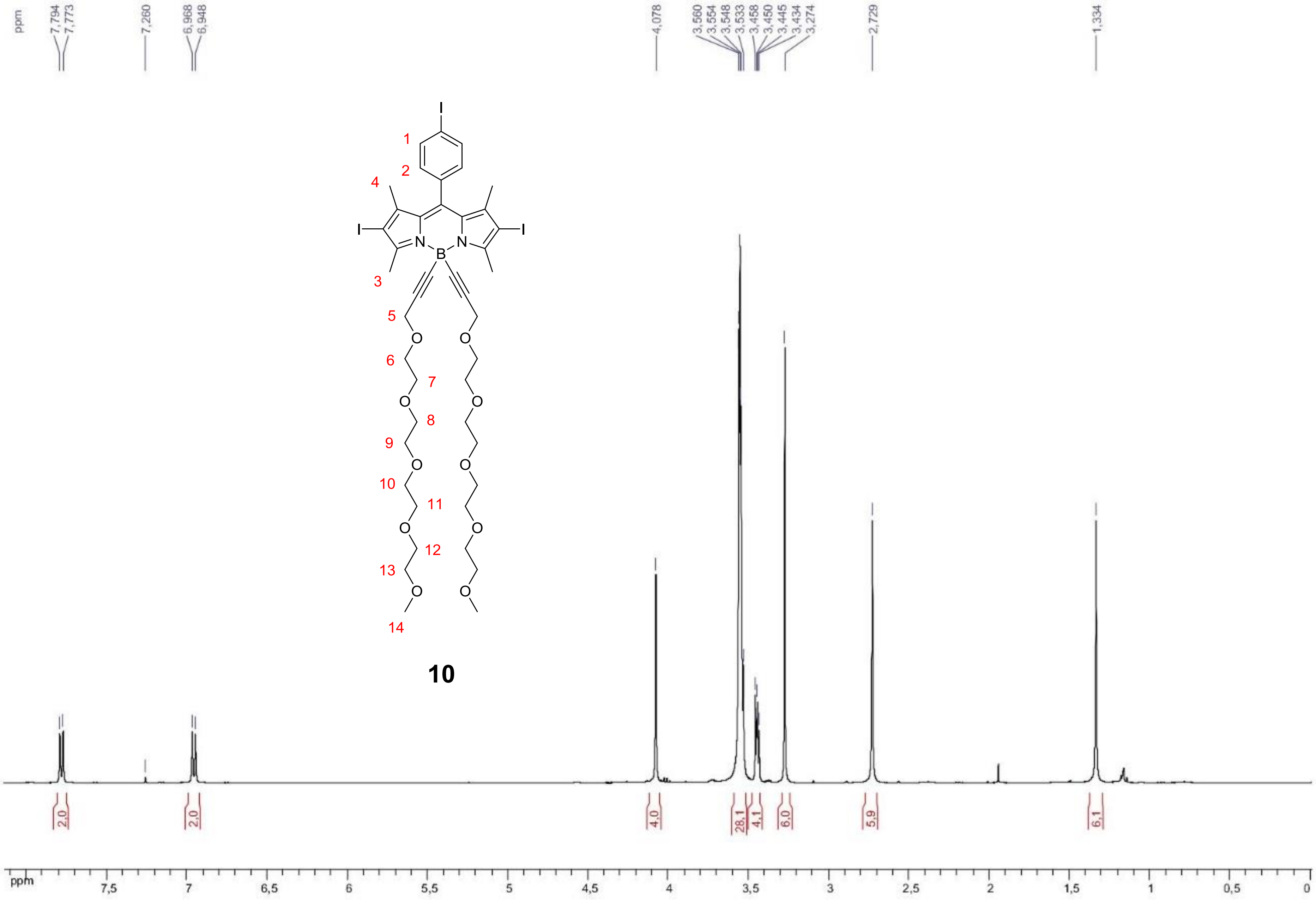

^{13}C-NMR spectrum ($CDCl_3$, 100 MHz) of compound 10:

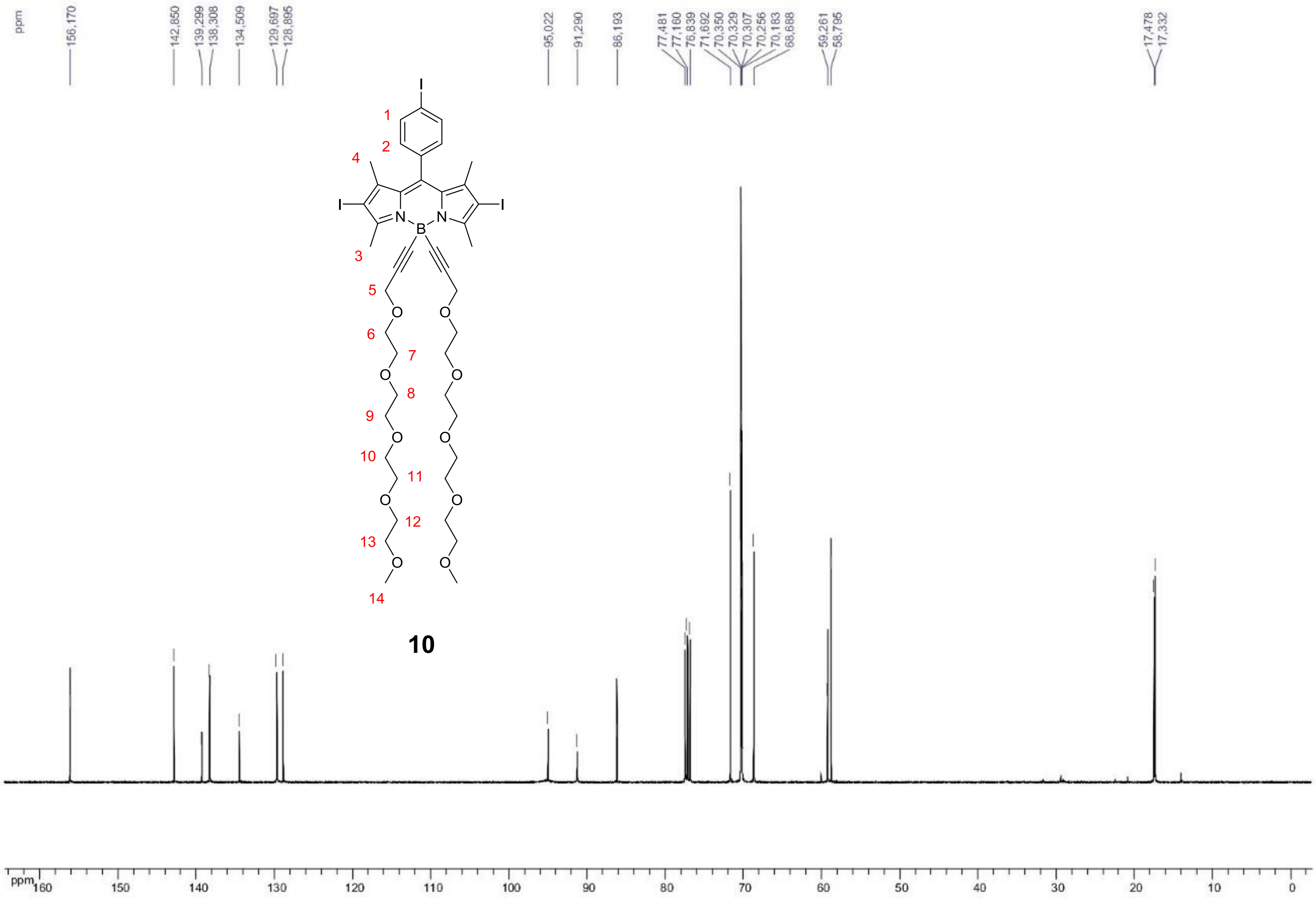

^{1}H-NMR spectrum ($CDCl_3$, 400 MHz) of compound 11:

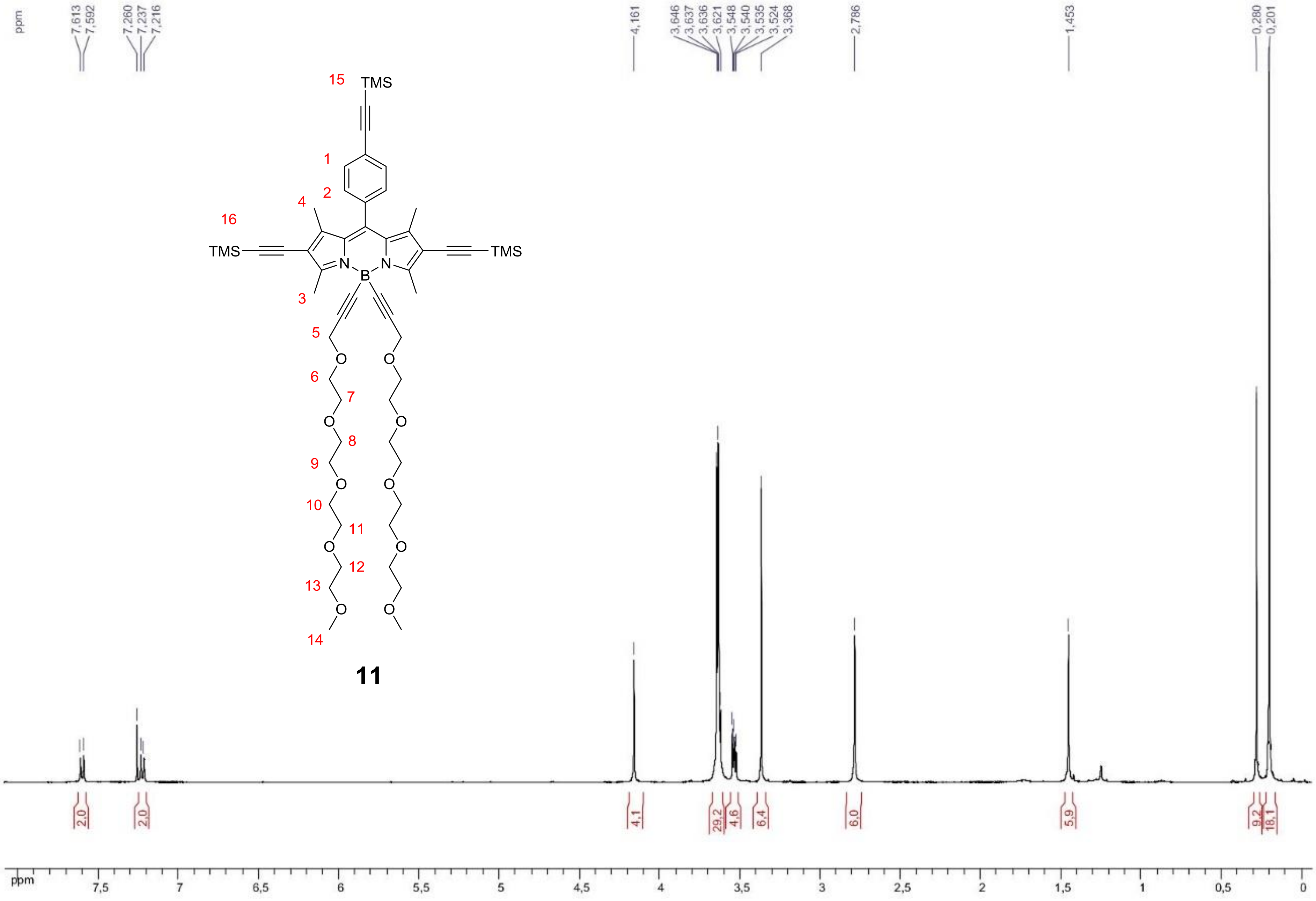

^{13}C-NMR spectrum ($CDCl_3$, 100 MHz) of compound 11:

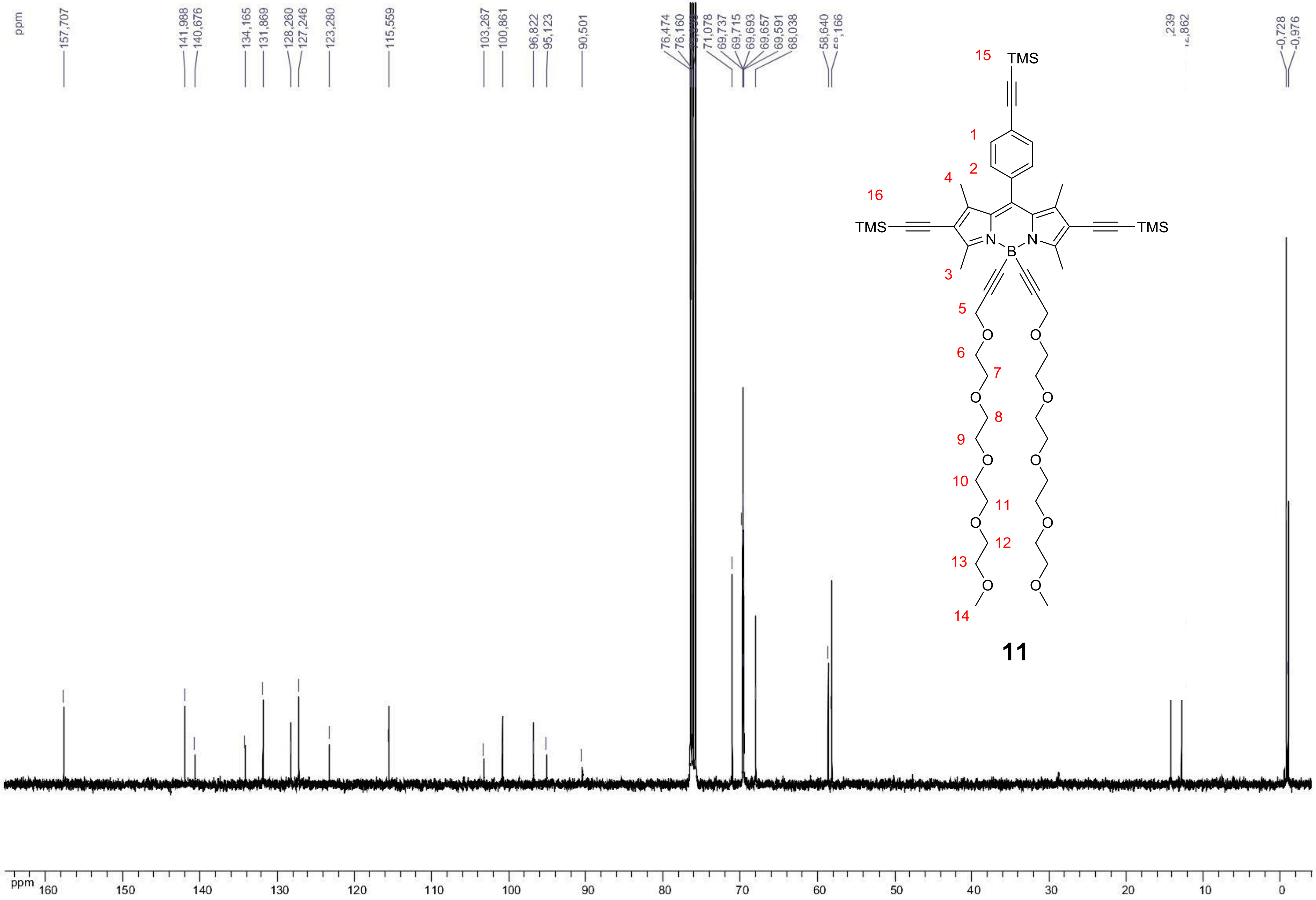

^{1}H-NMR spectrum ($CDCl_3$, 400 MHz) of compound 6b:

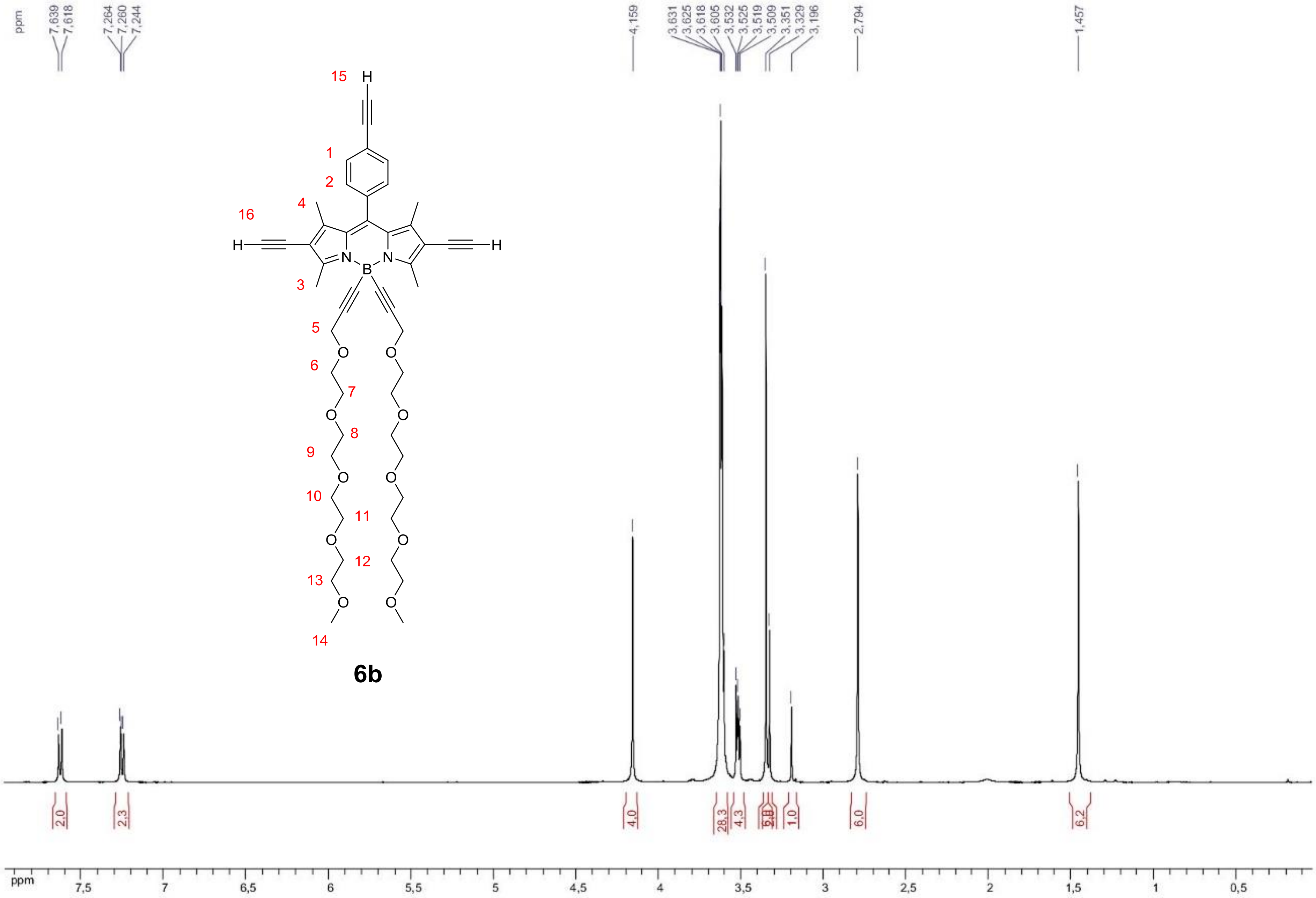

^{13}C-NMR spectrum ($CDCl_3$, 100 MHz) of compound 6b:

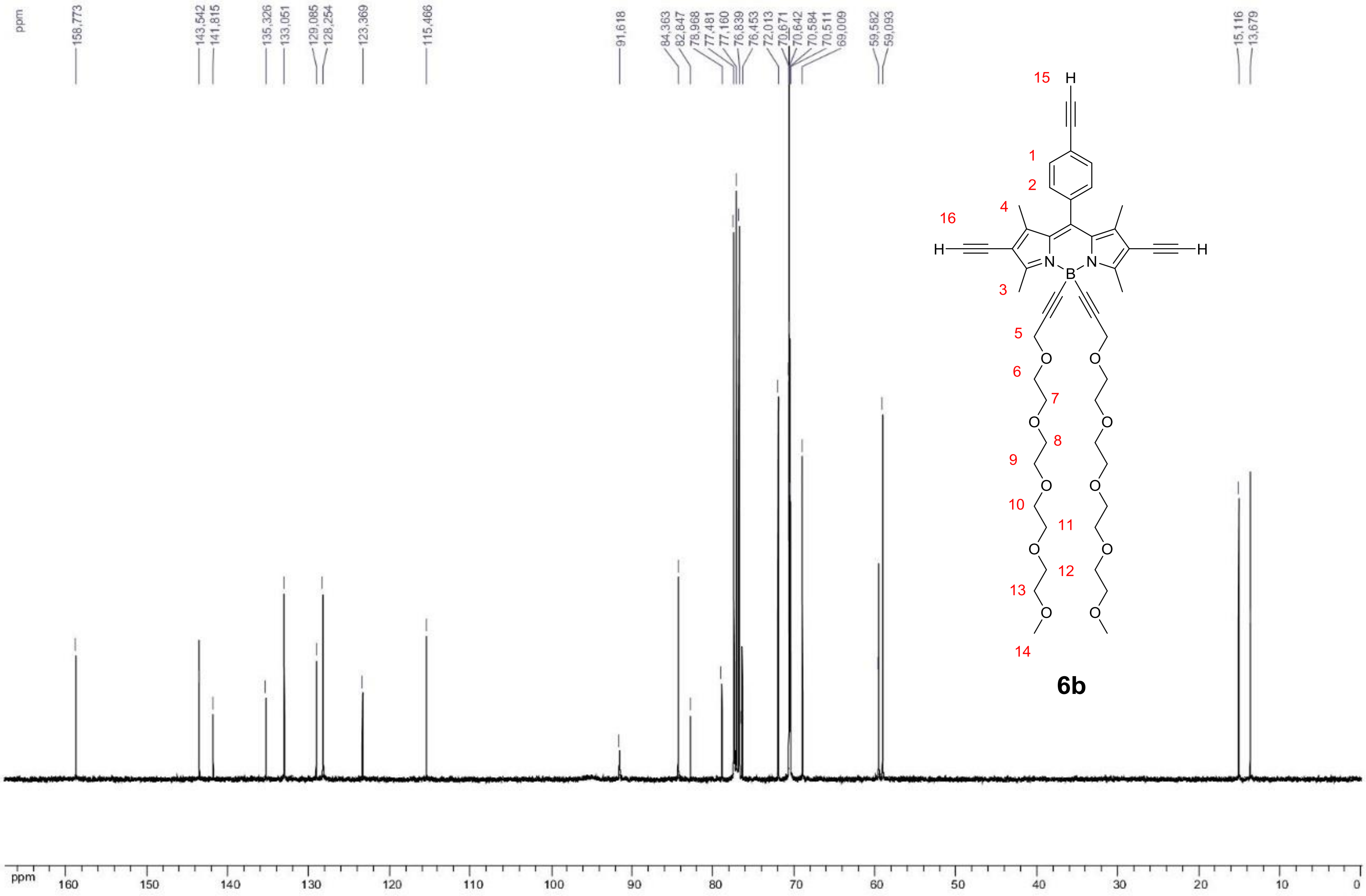

^{1}H-NMR spectrum ($CDCl_3$, 400 MHz) of compound 12a:

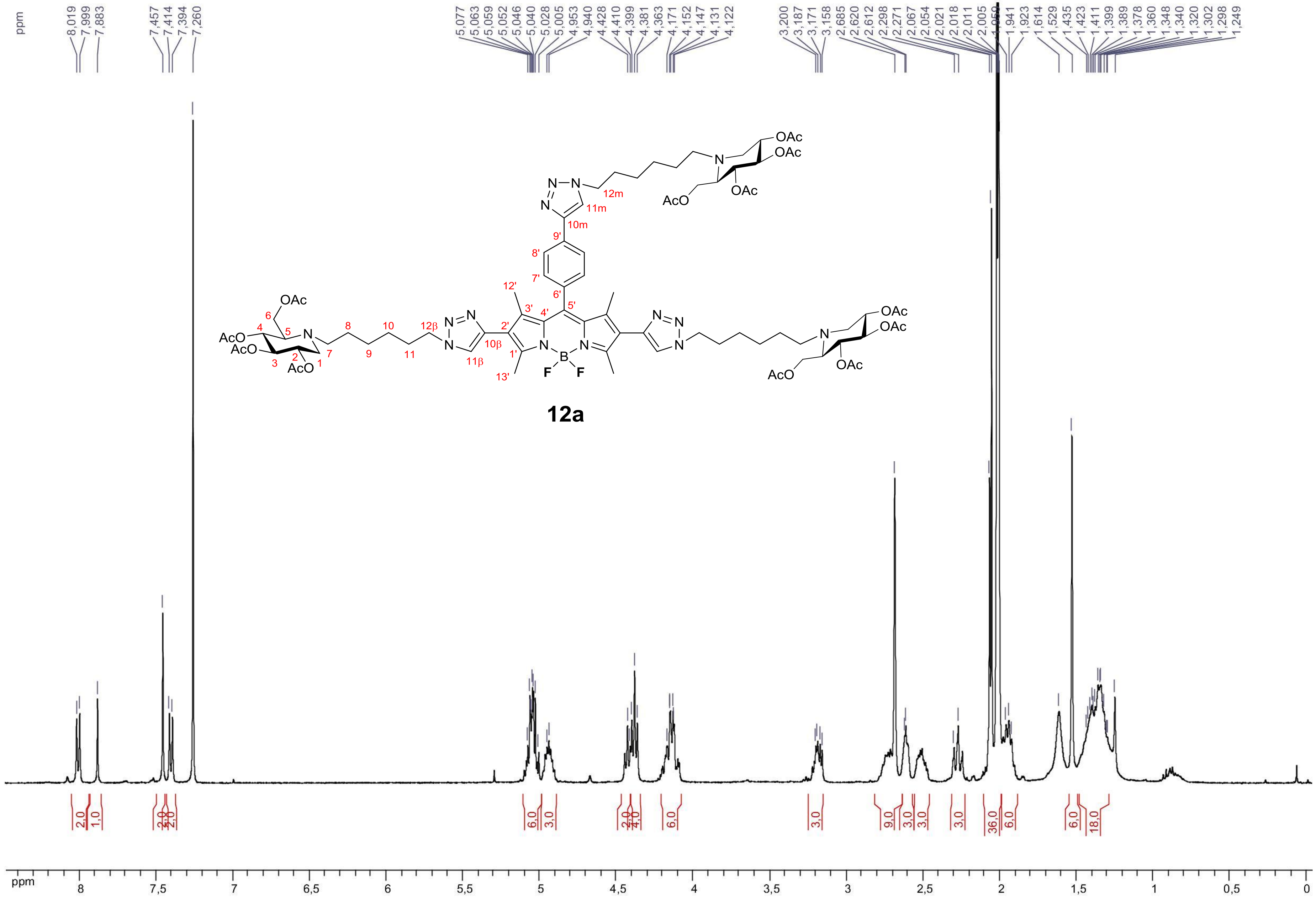

n

^{13}C-NMR spectrum ($CDCl_3$, 100 MHz) of compound 12a:

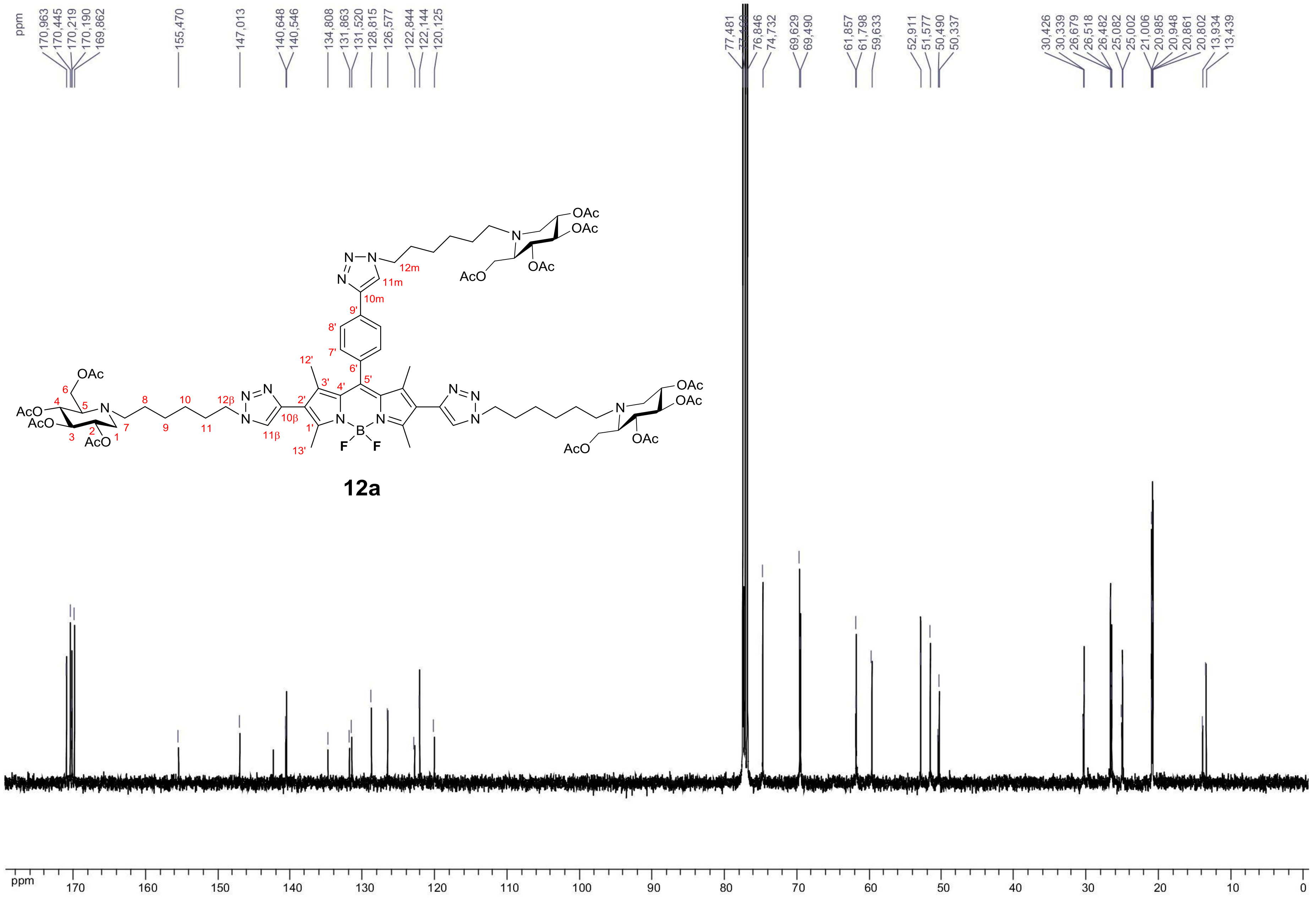

^{1}H-NMR spectrum ($CDCl_3$, 400 MHz) of compound 12b:

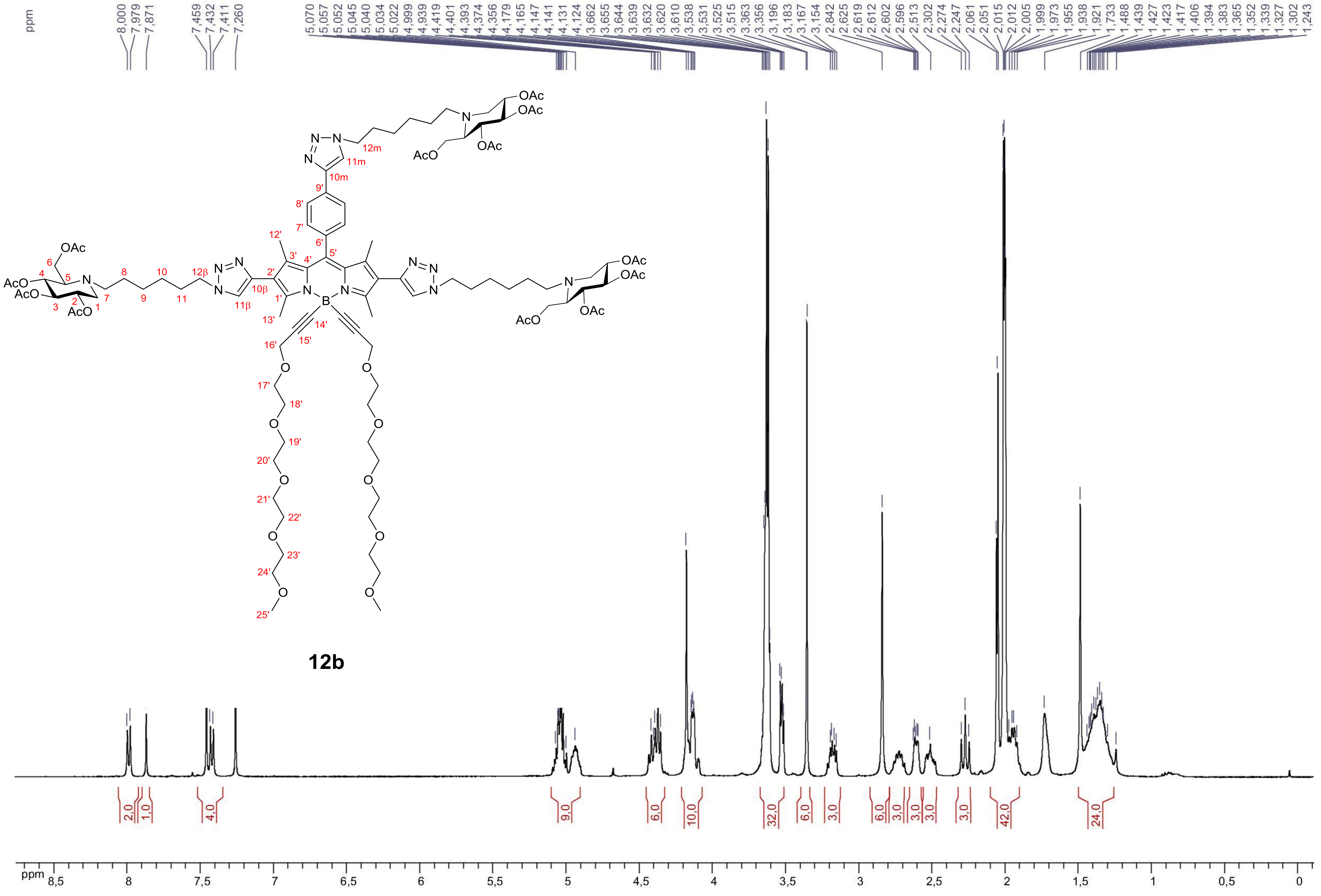

^{13}C-NMR spectrum ($CDCl_3$, 100 MHz) of compound 12b:

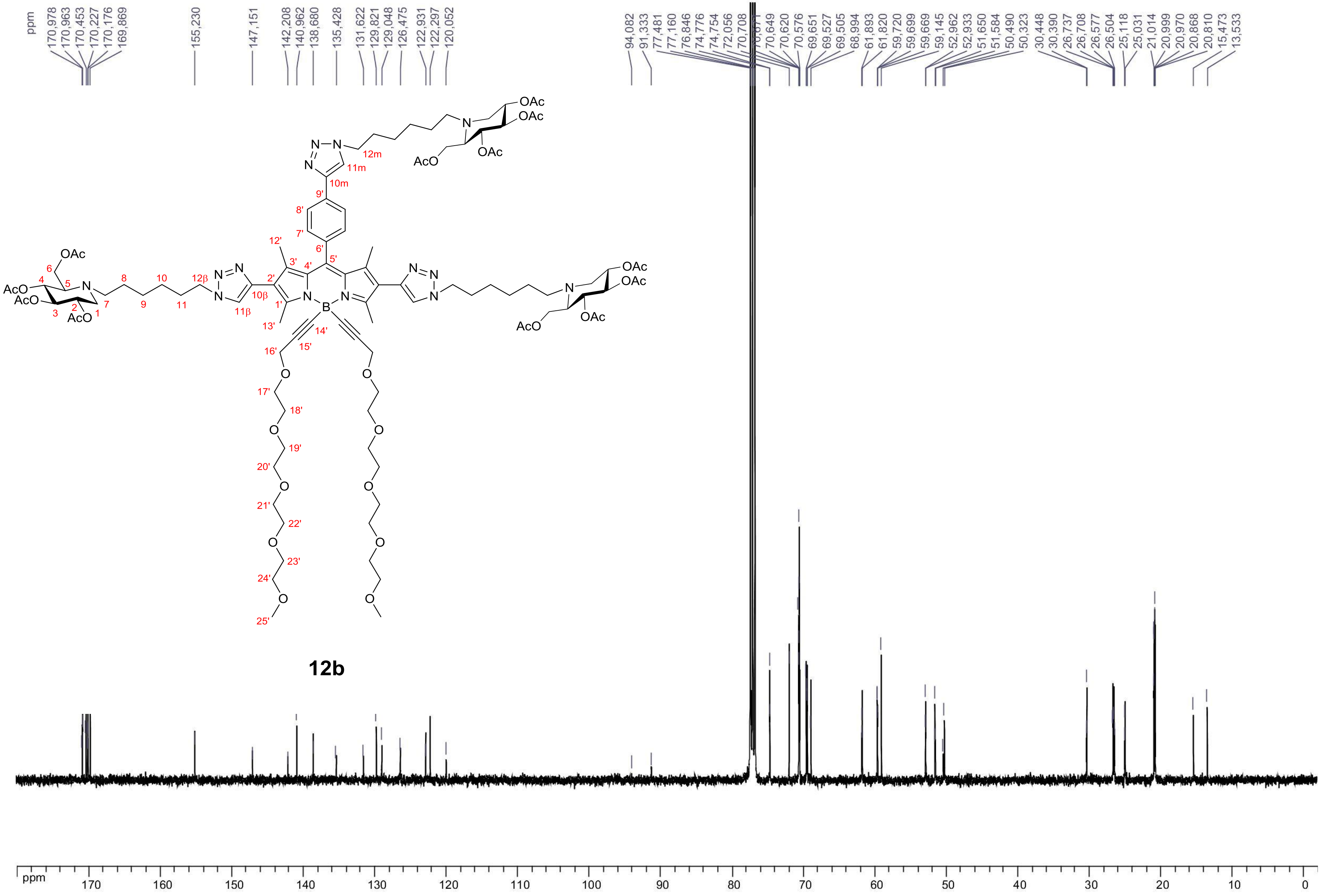

^{1}H-NMR spectrum (CD_3OD, 400 MHz) of compound 13a:

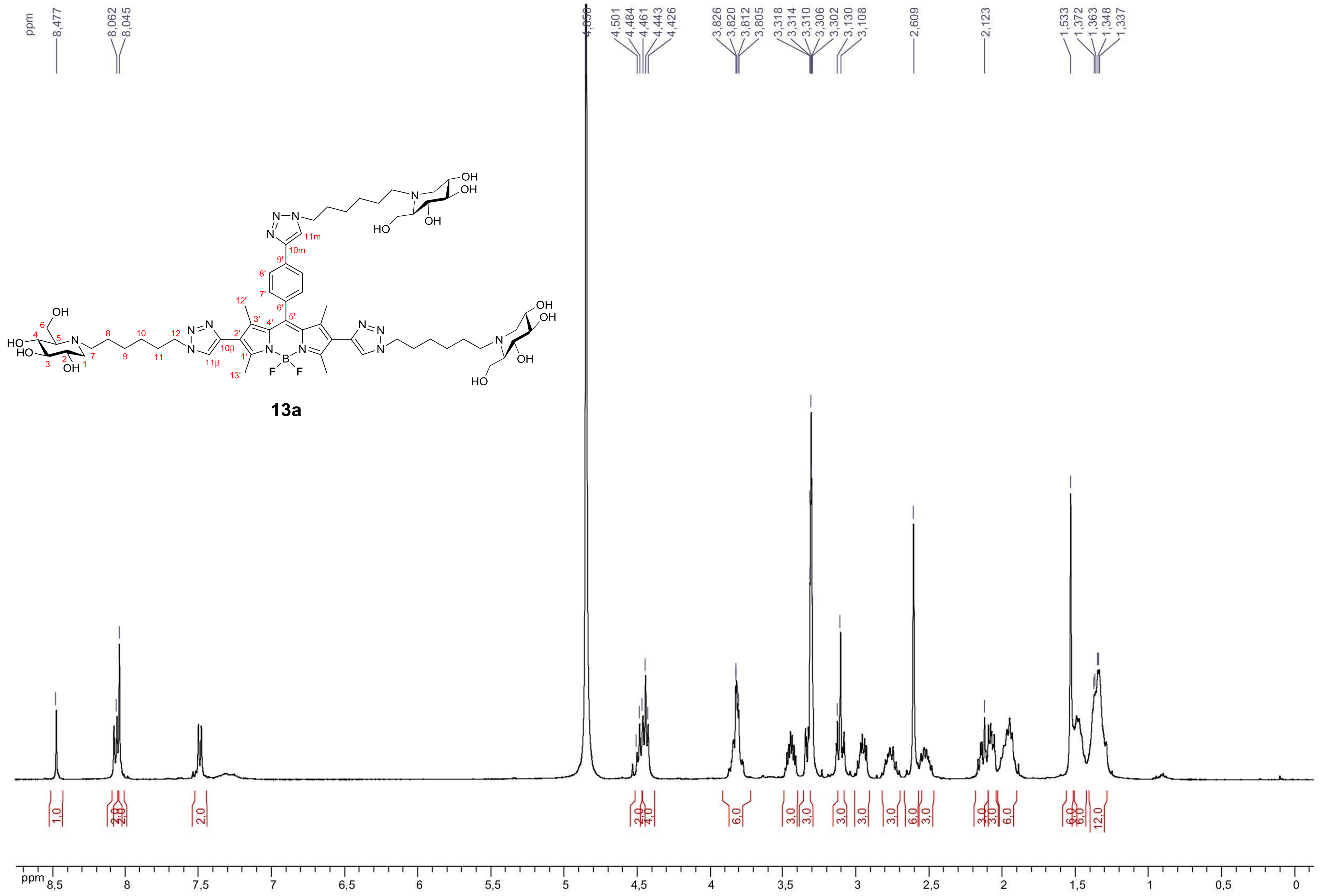

^{13}C-NMR spectrum (CD_3OD, 100 MHz) of compound 13a:

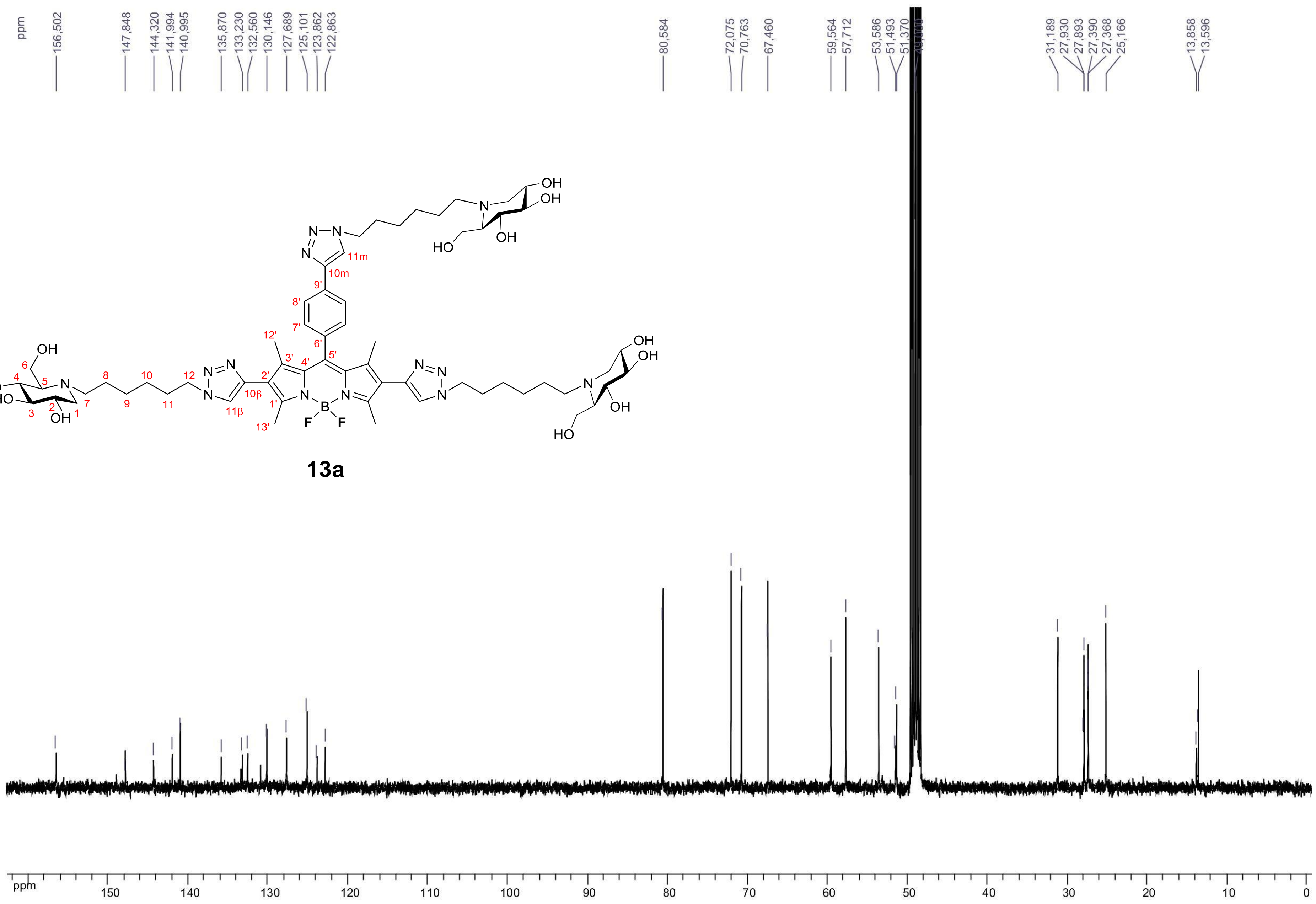

^{1}H-NMR spectrum (CD_3OD, 400 MHz) of compound 13b:

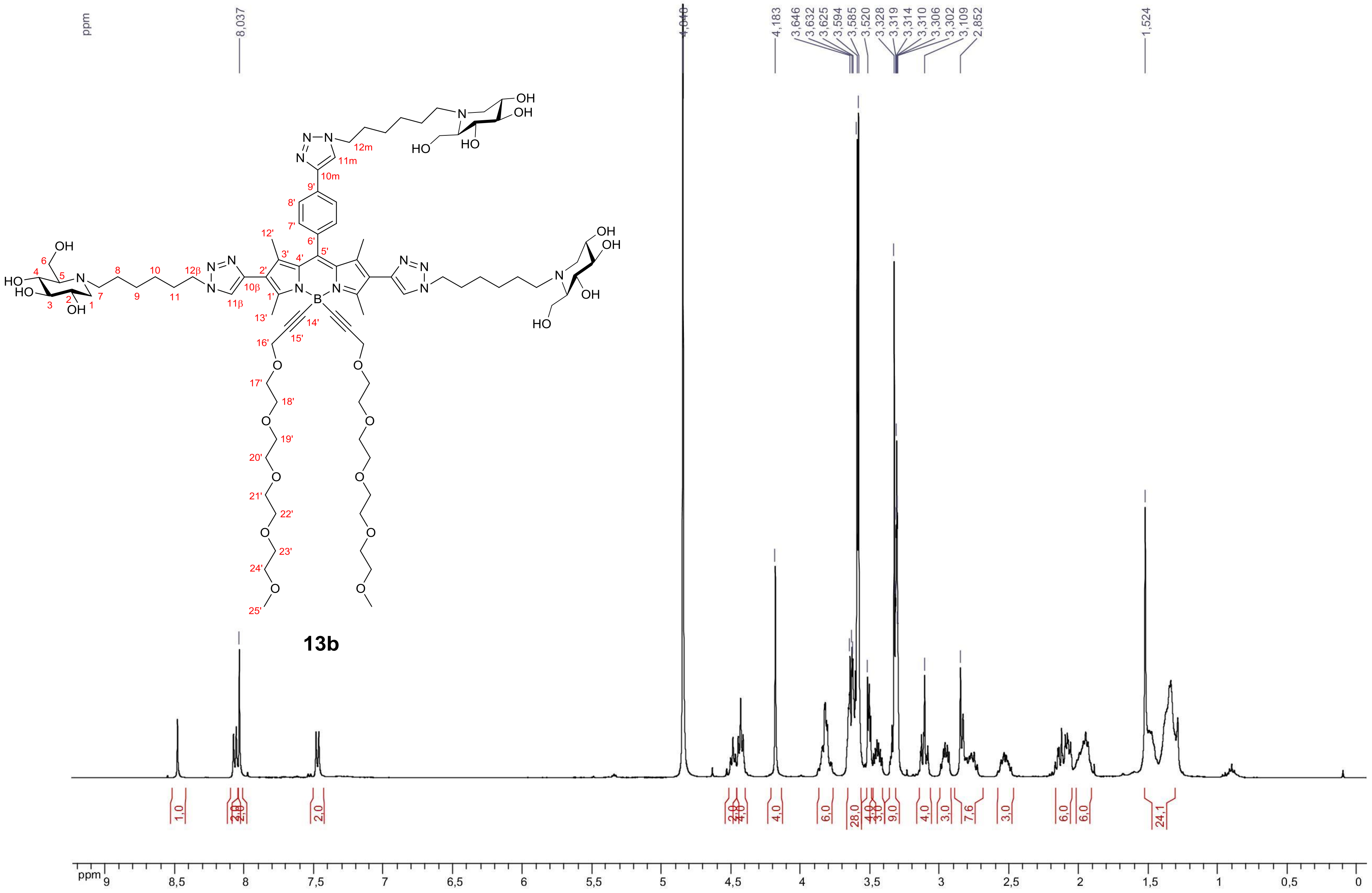

^{13}C-NMR spectrum (CD_3OD, 100 MHz) of compound 13b:

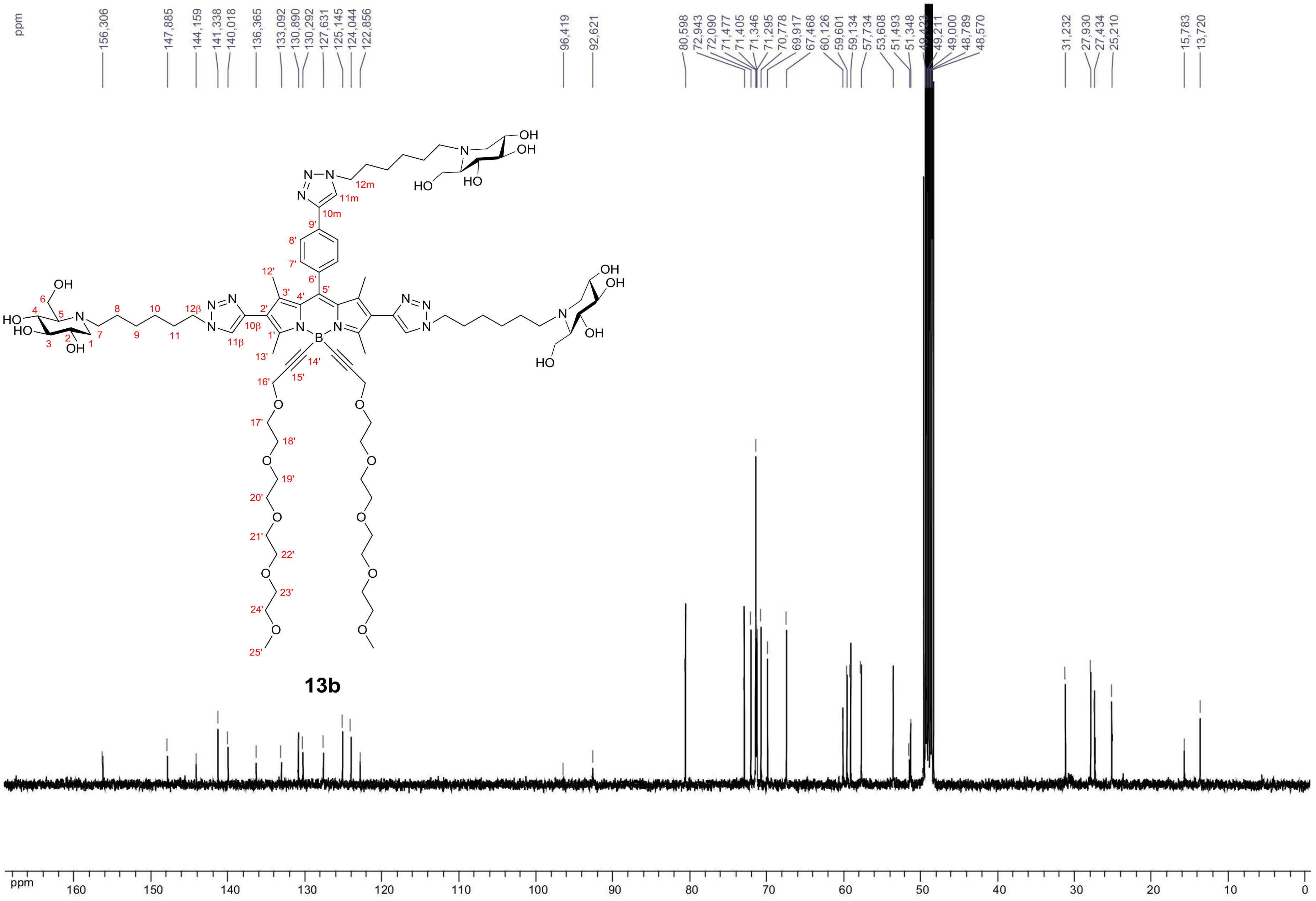

^{1}H-NMR spectrum ($CDCl_3$, 400 MHz) of compound 14a:

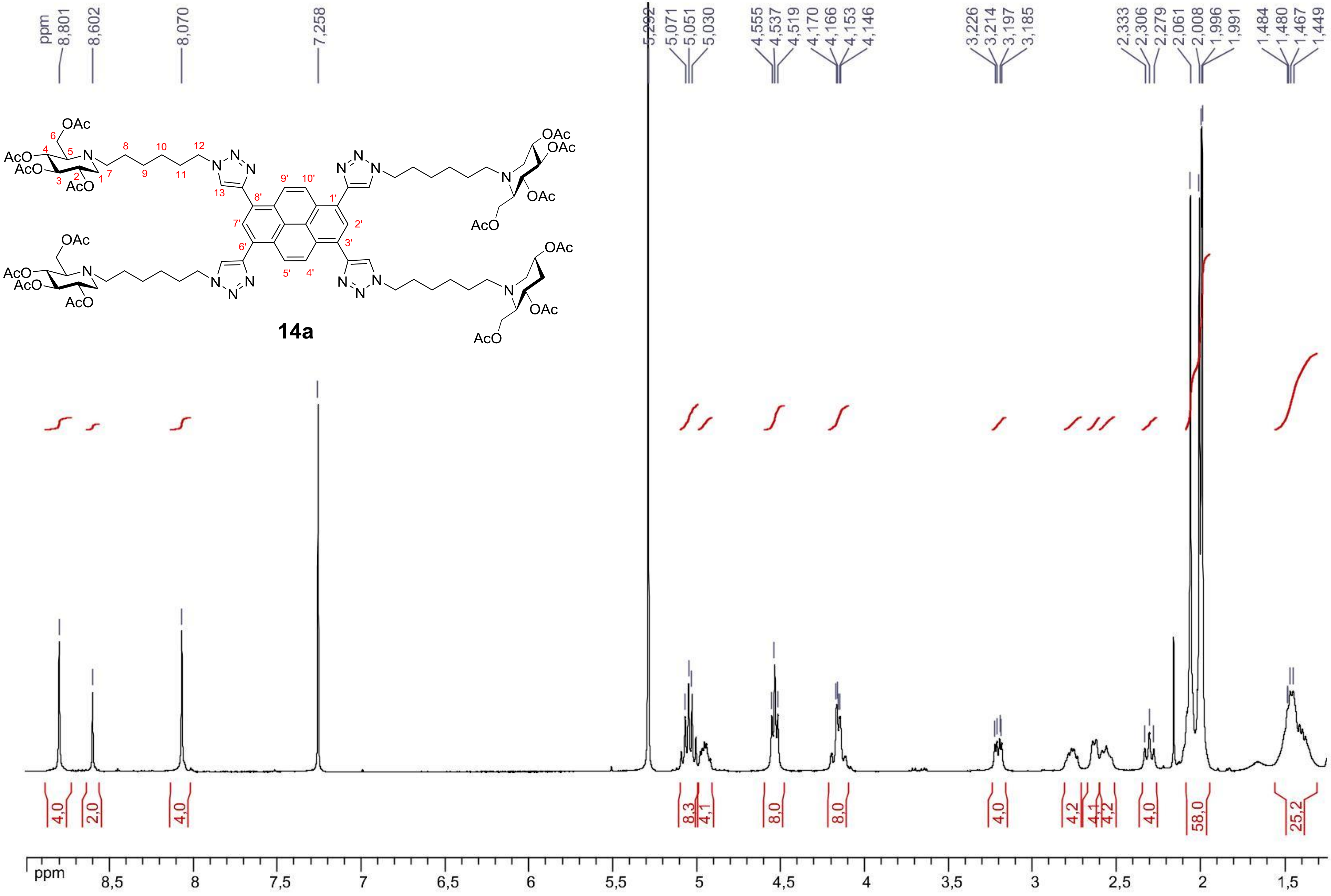

^{13}C-NMR spectrum ($CDCl_3$, 100 MHz) of compound 14a:

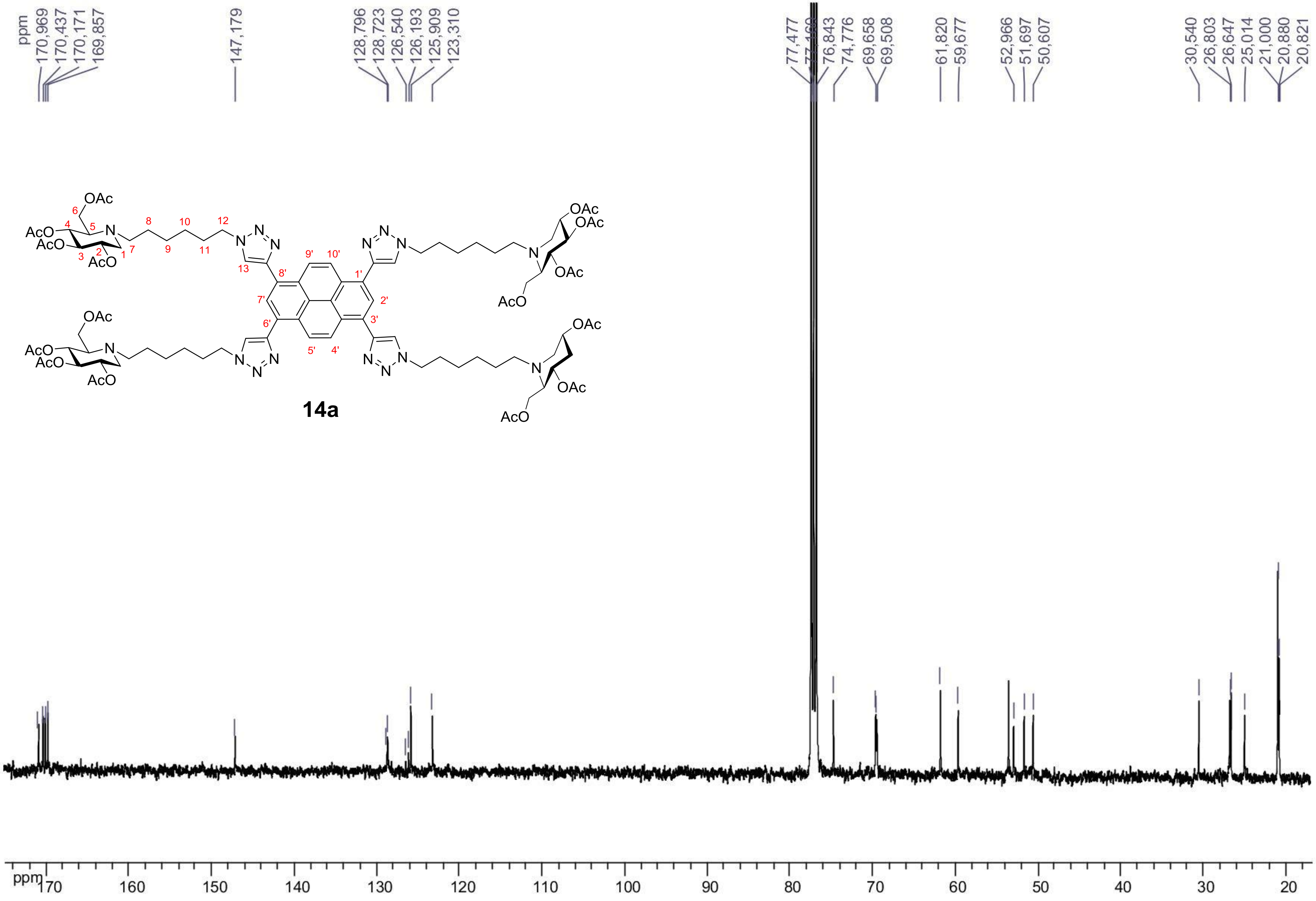

^{1}H-NMR spectrum ($CDCl_3$, 400 MHz) of compound 14b:

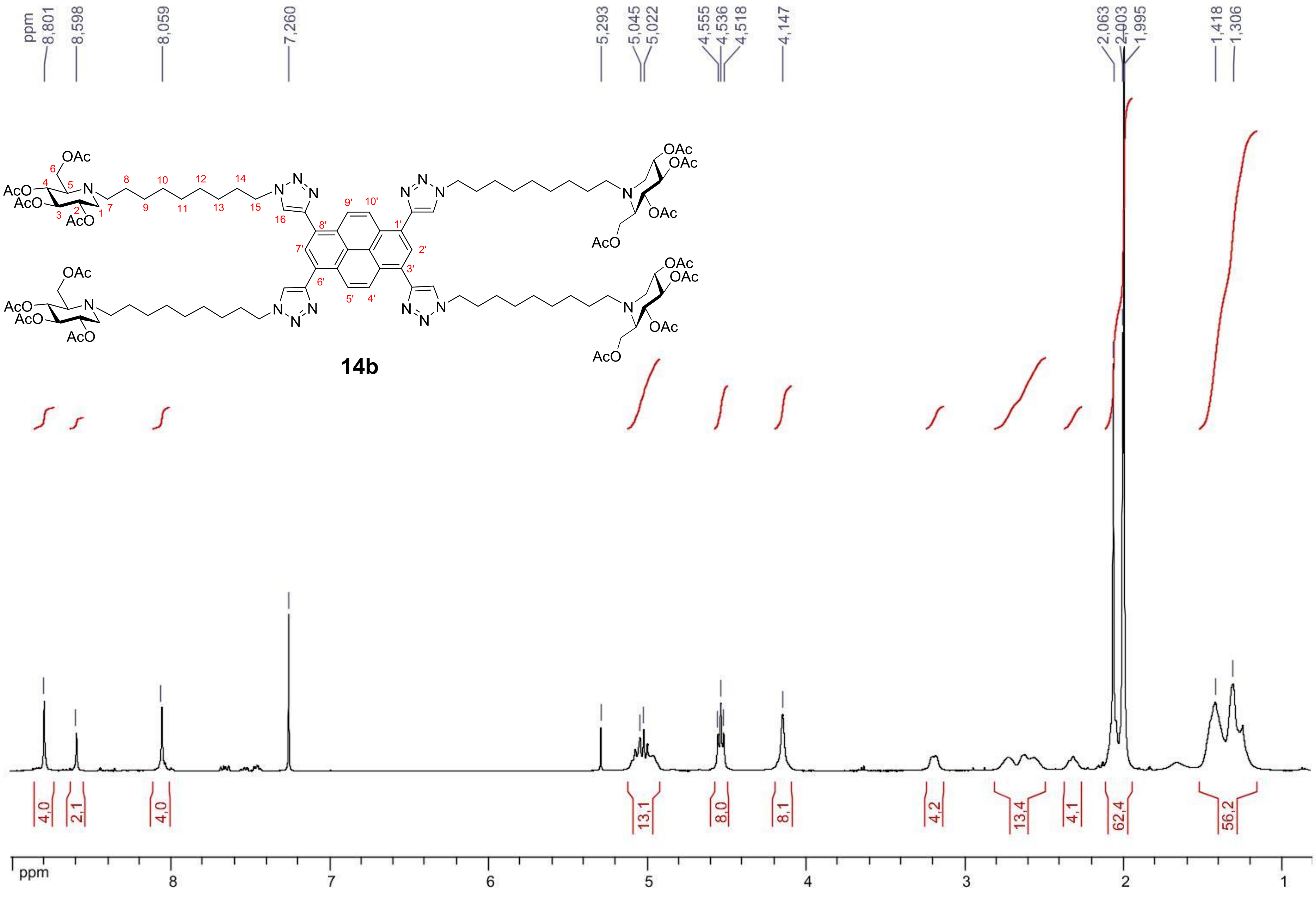

^{13}**C-NMR spectrum ($CDCl_3$, 100 MHz) of compound 14b:**

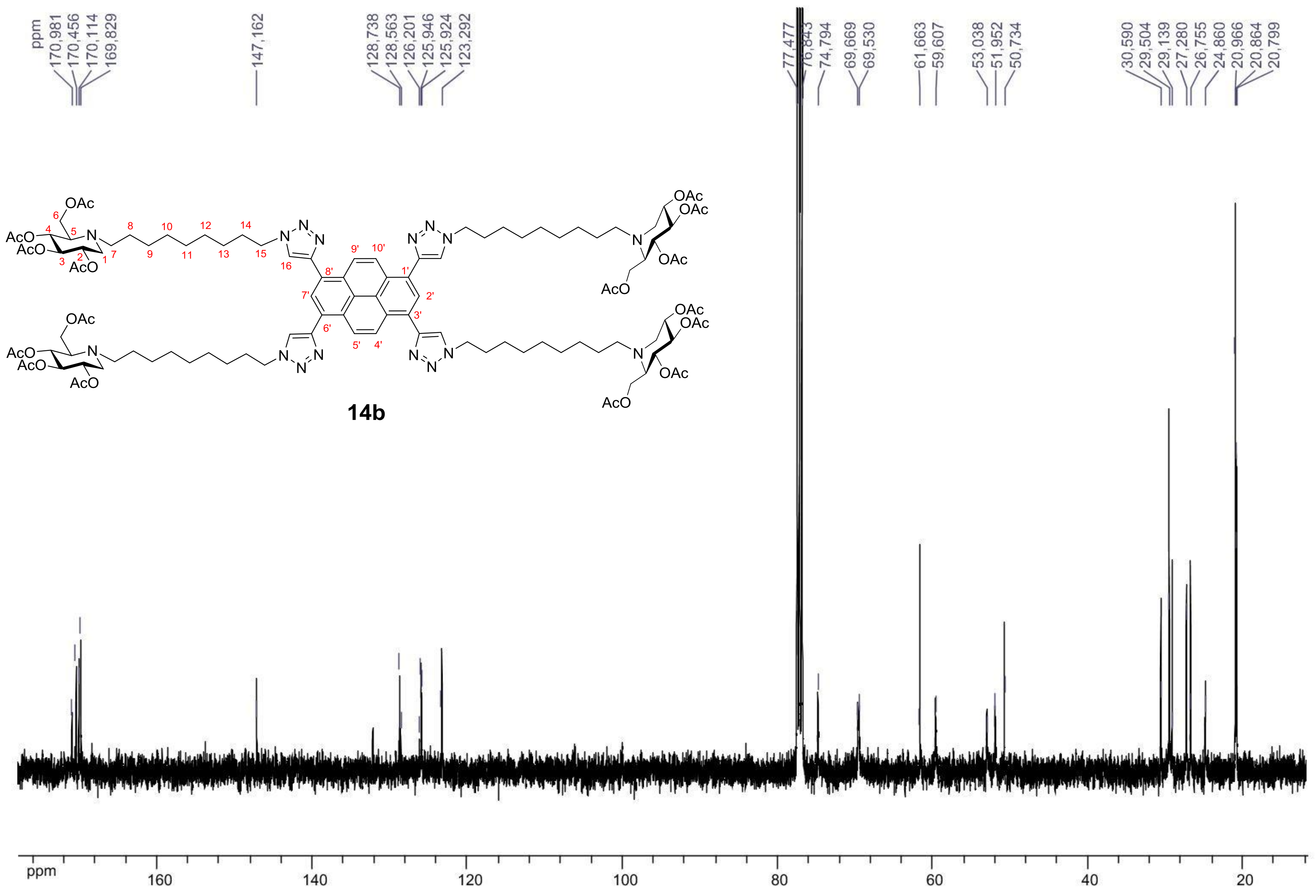

^{1}H-NMR spectrum (CD_3OD /$CDCl_3$/D_2O 4:1:1, 400 MHz) of compound 15a:

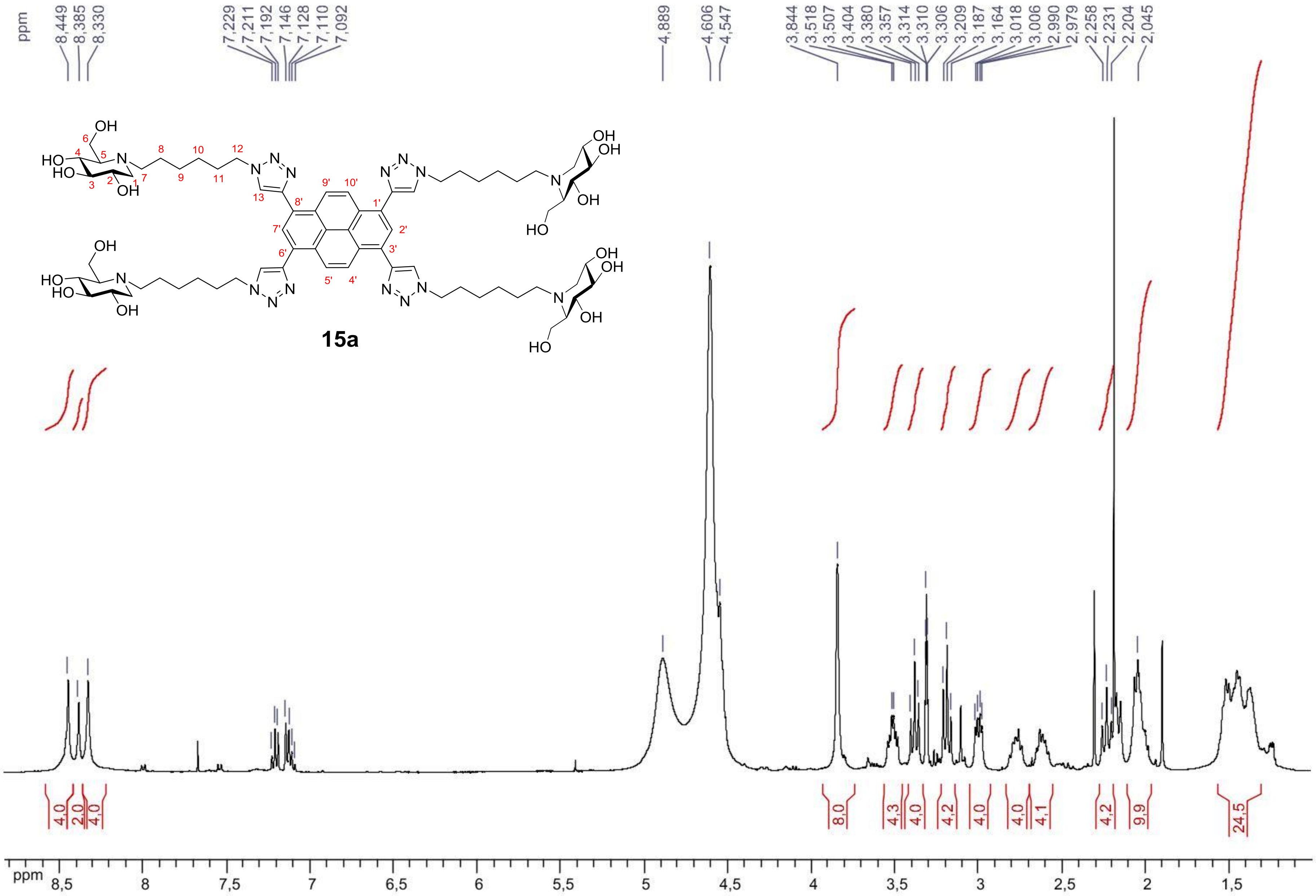

^{13}C-NMR spectrum ($CD_3OD/CDCl_3/D_2O$ 4:1:1, 100 MHz) of compound 15a:

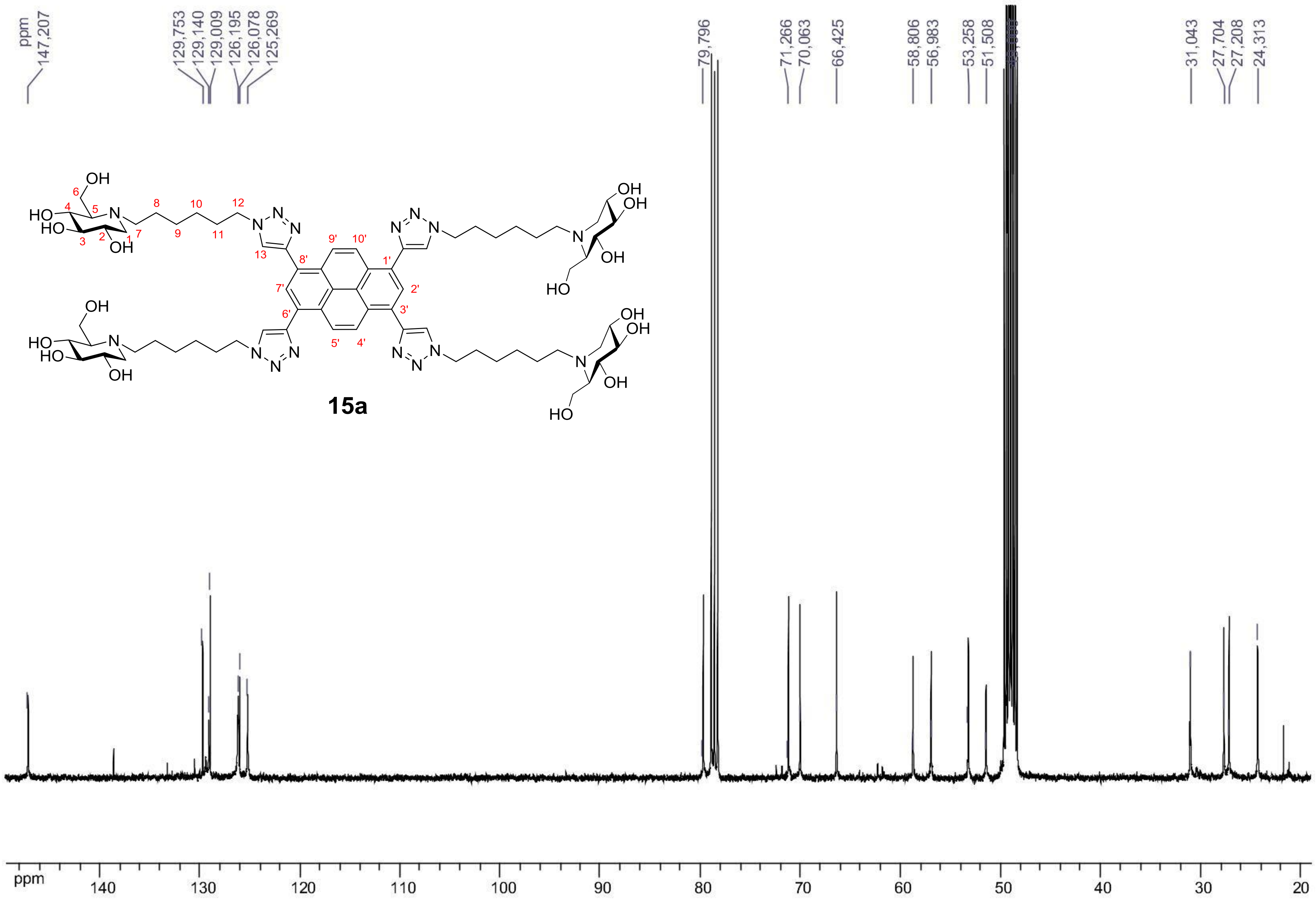

^{1}H-NMR spectrum (CD_3OD /$CDCl_3$/D_2O 4:1:1, 400 MHz) of compound 15b:

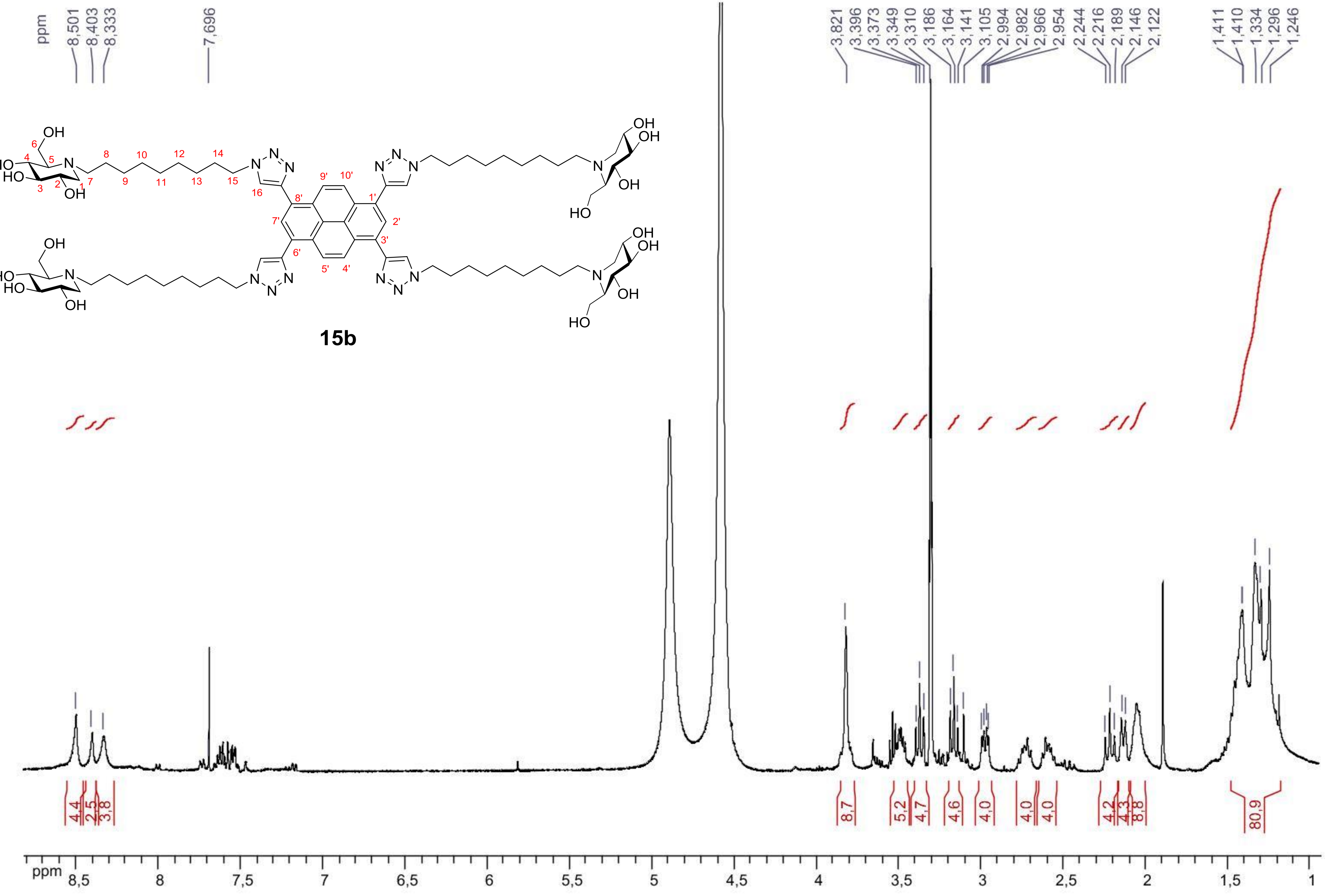

^{13}C-NMR spectrum (CD_3OD /$CDCl_3$/D_2O 4:1:1, 100 MHz) of compound 15b:

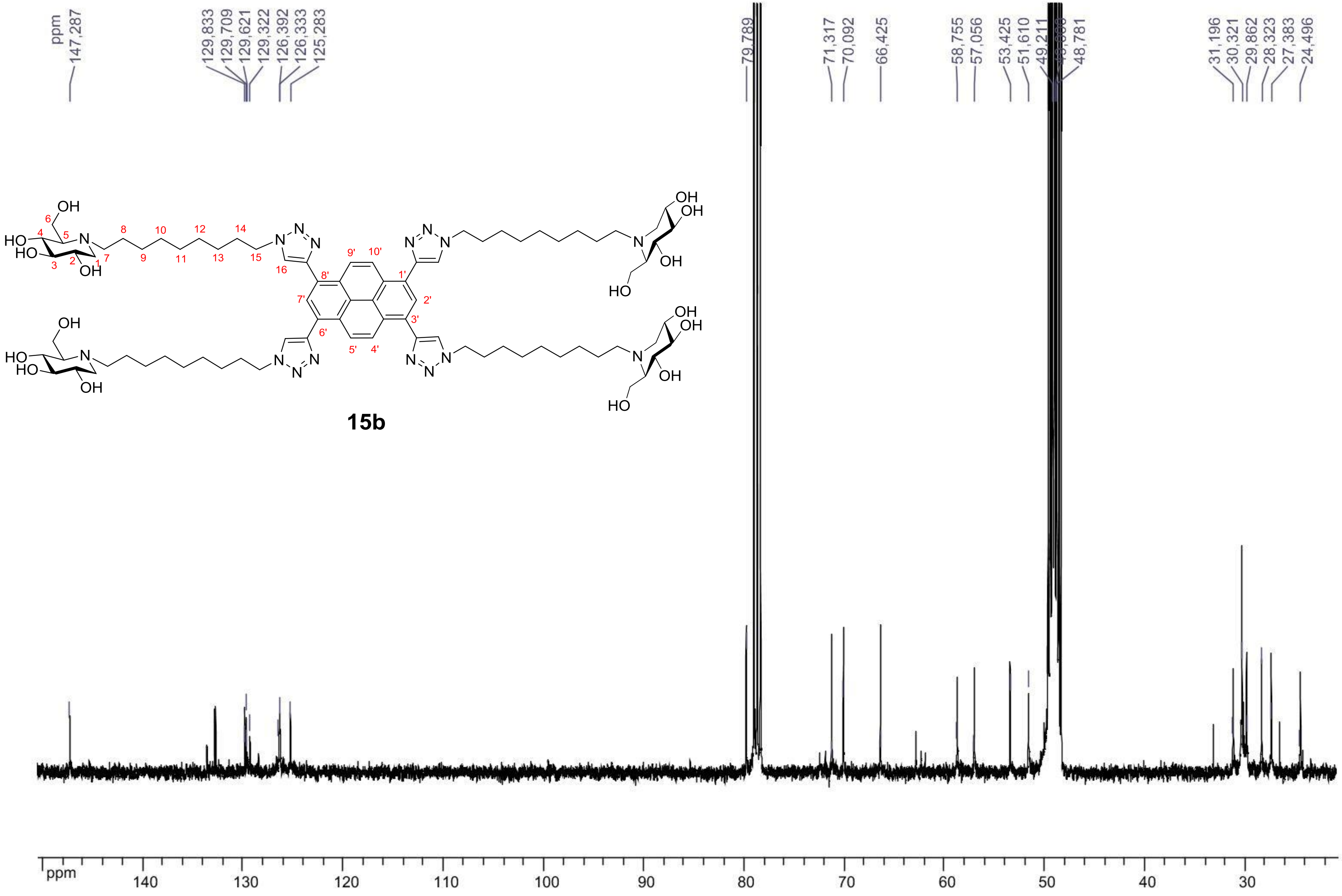

Supplement: File 1 — Characterization data and NMR spectra of all new compounds. [file Beilstein_J_Org_Chem-11-659-s001.pdf]
